# Targeting TPC2 sensitizes acute lymphoblastic leukemia cells to chemotherapeutics by impairing lysosomal function

**DOI:** 10.1038/s41419-022-05105-z

**Published:** 2022-08-01

**Authors:** Franz Geisslinger, Martin Müller, Yu-Kai Chao, Christian Grimm, Angelika M. Vollmar, Karin Bartel

**Affiliations:** 1 Ludwig-Maximilians University, Departement of Pharmacy, Pharmaceutical Biology, Munich, Germany; 2 Ludwig-Maximilians University, Walther-Straub-Institute of Pharmacology and Toxicology, Munich, Germany

**Keywords:** Acute lymphocytic leukaemia, Acute lymphocytic leukaemia, Apoptosis

## Abstract

Despite novel therapy regimens and extensive research, chemoresistance remains a challenge in leukemia treatment. Of note, recent studies revealed lysosomes as regulators of cell death and chemotherapy response, suggesting this organelle is a novel target for chemosensitization. Interestingly, drug-resistant VCR-R CEM acute lymphoblastic leukemia (ALL) cells have an increased expression of the lysosomal cation channel Two-Pore-Channel 2 (TPC2) compared to drug-naïve CCRF-CEM ALL cells. Concurrently, knockout (KO) of TPC2 sensitized drug-resistant VCR-R CEM cells to treatment with cytostatics. The chemosensitizing effect could be confirmed in several cell lines as well as in heterogeneous, patient-derived xenograft ALL cells, using the pharmacological TPC2 inhibitors naringenin and tetrandrine. We reveal that a dual mechanism of action mediates chemo sensitization by loss of lysosomal TPC2 function. First, because of increased lysosomal pH, lysosomal drug sequestration is impaired, leading to an increased nuclear accumulation of doxorubicin and hence increased DNA damage. Second, lysosomes of TPC2 KO cells are more prone to lysosomal damage as a result of morphological changes and dysregulation of proteins influencing lysosomal stability. This leads to induction of lysosomal cell death (LCD), evident by increased cathepsin B levels in the cytosol, truncation of pro-apoptotic Bid, as well as the reversibility of cell death by co-treatment with the cathepsin B inhibitor CA-074Me in TPC2 KO cells. In summary, this study establishes TPC2 as a novel, promising, druggable target for combination therapy approaches in ALL to overcome chemoresistance, which could be exploited in the clinic in the future. Additionally, it unravels LCD signaling as an important death-inducing component upon loss of TPC2 function.

## Introduction

Leukemia is a heterogeneous disease defined as cancer of the early blood-forming cells, which is among the top 10 causes of cancer-related deaths worldwide [1]. Among different sub-classifications of leukemia, acute lymphoblastic leukemia (ALL) is characterized by a rapid pathologic proliferation of degenerated lymphocyte precursors [2].

The main treatment strategy for ALL is chemotherapy. Despite the development of many innovative targeted therapy strategies, like inhibition of cancer-specific kinases and immunotherapy, classic cytostatics, such as vincristine and doxorubicin, are still an indispensable part of common ALL therapy regimens [3]. Unfortunately, the development of resistance against these cytostatics is strongly pronounced and frequently occurs [4], causing a challenge in clinical cancer therapy [5].

Interestingly, lysosomes have come into focus of cancer research lately and might provide a promising target to overcome chemoresistance. Lysosomes are intracellular organelles with an acidic lumen, separated from the cytosol by a lipid bilayer [6]. Besides the widely known implication of lysosomes in macromolecule digestion, the endolysosomal system emerged as an interesting signaling hub regarding nutrient homeostasis [7], autophagy [8], and regulation of cell death [9]. Nowadays it is well-known that lysosomes are implicated in a cell death pathway called lysosomal cell death (LCD), characterized by induction of selective defects in the lysosomal membrane, which cause lysosomal contents, most importantly cathepsins, to leak into the cytosol and induce cell death [9].

Given their broad regulatory role, it has become evident now, that lysosomal function can immensely influence response to chemotherapy. For instance, because of their acidic pH, weak base cytostatics accumulate within lysosomes, where they get protonated and subsequently trapped inside the lysosomal lumen, unable to reach their intracellular targets [10, 11]. Despite several aspects of the lysosomal role in chemoresistance being known, cancer-specific approaches targeting their function to reverse chemoresistance are scarce to date.

One promising approach to manipulate lysosomal function with a certain degree of cancer specificity is targeting its membrane-abundant proteins, such as transporters and ion channels. For instance, inhibition of the lysosomal V-ATPase in cancer is of great interest as a potential therapeutic strategy [12, 13]. Additionally, different families of lysosomal cation channels have been reported to play essential roles in lysosome-connected processes. Concurrently, these channels gain increasing interest as potential anti-cancer targets. One of these ion channels, which is in the focus of current research, is Two-Pore Channel 2 (TPC2), a lysosome-abundant cation channel permeable for sodium and calcium [14, 15]. It has been proposed as a regulator of vesicular pH and autophagy [16]. Importantly, TPC2 has already been associated with cancer progression in vitro and in vivo [17–19]. It has been shown, that high TPC2 expression is correlated with poor survival in ovarian cancer, as well as head and neck cancer (Human protein atlas). Furthermore, mutations in TPC2 are reported in cancer patients, with a high mutation rate in patients suffering from ALL (NCI GDC Data portal) [20]. Yet, large studies on the role of TPC2 in ALL are still missing.

Based on the findings that chemoresistance is a major problem in ALL, the importance of lysosomes for chemoresistance, and the identification of TPC2 as a potentially poor survival factor, we generated our research hypothesis. We suggest that impairing lysosomal function by manipulation of TPC2 might be a reasonable strategy to reverse lysosome-mediated chemoresistance and we elucidate the underlying mechanisms of action.

## Materials and Methods

### Cell lines and culture

VCR-R CEM and CCRF-CEM cells were obtained from Prof. Maria Kavallaris (University of New South Wales, Sydney, Australia) [21] and were cultured in RPMI 1640 (PAN Biotech, Aidenbach, Germany) containing 10% FCS (PAN Biotech). VCR-R CEM cells are a multidrug-resistant subline of CCRF-CEM cells [22], generated by long-term exposure to increasing concentrations of vincristine [23]. Jurkat cells were bought from ATCC and cultured in RPMI 1640 containing 10% FCS and pyruvate. Cell lines were typically passaged twice to thrice a week. HeLa cells were purchased from DSMZ (Braunschweig, Germany) and cultured in DMEM (PAN Biotech) containing 10% FCS. The model of ALL patients’ leukemia cells growing in mice has been described previously [24]. In the present study, patient-derived xenograft (PDX) cells were engrafted and freshly isolated from the bone marrow or spleen of NSG mice (The Jackson Laboratory, Bar Harbour, ME, USA) and subsequently cultured in RPMI 1640 supplemented with 20% FCS and P/S. Peripheral blood mononuclear cells (PBMC) were obtained from ATCC and cultured in RPMI 1640 supplemented with 20% FCS and P/S.

Cell line STR profiling was performed. None of the cell lines used are listed in the database of commonly misidentified cell lines maintained by ICLAC. All cells are proven to be mycoplasma-free quarterly.

### Compounds

Doxorubicin (10 mM in water), topotecan (10 mM in water), naringenin (100 mM in DMSO), chloroquine (100 mM in water), and 5-fluorouracil (50 mM in water) were purchased from Sigma Aldrich (Taufkirchen, Germany). Vincristine (100 mM in DMSO) was purchased from Adipogen (San Diego, CA, USA). Tetrandrine (10 mM in DMSO) was purchased from Sigma Aldrich and purified by the group of Prof. Franz Bracher (Ludwig-Maximilians University, Munich). CA-074Me (50 mM in DMSO) was purchased from Selleckchem (Houston, TX, USA).

### Quantitative real-time PCR

RNA was isolated from cellular samples using the RNeasy Mini Kit (Qiagen) as described by the manufacturer. Cells were collected by centrifugation, washed twice in ice-cold PBS, and resuspended in Buffer RLT containing 40 µM DTT. The resulting mRNA yield was determined on a Nanodrop Spectrophotometer. 1250 ng RNA were reversely transcribed using the High-Capacity cDNA Reverse Transcription Kit (Applied Biosystems, Waltham, MA, USA) according to the manufacturer’s instructions. RT-qPCR was conducted using PowerUp™ SYBR® Green Master Mix (Applied Biosystems) (2 µl cDNA (=50 ng), 6.25 µl PowerUp™ SYBR® Green Master Mix, 3.25 µl RNAse-free water, 0.5 µl (200 nM) forward and reverse primer/well). All measurements were conducted using the QuantStudio™ 3 Real-Time PCR System (Applied Biosystems) and results were evaluated using the ΔΔCT method as described previously [25]. Actin served as a housekeeping gene. Primers (Table 1) were purchased from Metabion (Planegg, Germany) and validated for their efficiency.

**Table 1:** Primers for qPCR.

Target	Primer	Sequence (5'-3')
ABCB1	FW	CAG CTG TTG TCT TTG GTG CC
ABCB1	RV	GTC TGG CCC TTC TTG ACC TC
Actin	FW	CCA ACC GCC AGA AGA TGA
Actin	RV	CCA GAG GCG TAC AGG GAT AG
ATP6V0C	FW	ATG CTT CGT TTT TCG CCG TC
ATP6V0C	RV	ATG ACA GAC ATG GCC GCA A
CTSB	FW	CTG GCA GGT TGA AGT AGG GG
CTSB	RV	CCG CTA ATA ACG GCA GTT GC
CTSD	FW	GAC ATC CAC TAT GGC TCG GG
CTSD	RV	AGC ACG TTG TTG ACG GAG AT
LAMP1	FW	CGT CCT TGG GCG TCT CTA AT
LAMP1	RV	CAC AGC GCA GAA CAG GAT CA
NPC1	FW	TTC GGC AGC TTC AGA CAC TA
NPC1	RV	TTC AGT AGG TTA TAA AAA CAG GAT GG
TFEB	FW	CAA GGC CAA TGA CCT GGA C
TFEB	RV	AGC TCC CTG GAC TTT TGC AG
TPC1	FW	GGA GCC CTT CTA TTT CAT CGT
TPC1	RV	CGG TAG CGC TCC TTC AAC T
TPC2	FW	TGC ATT GAT CAG GCT GTG GT
TPC2	RV	GAA GCT CAA AGT CCG TTG GC
TRPML1	FW	TCT TCC AGC ACG GAG ACA AC
TRPML1	RV	GCC ACA TGA ACC CCA CAA AC
TRPML2	FW	AAC GGT GTT TCC TGT TCC GA
TRPML2	RV	GCC ATT GCA TTT CTG ACG GTT A
TRPML3	FW	TGC TTC TGT GGA TGG ATC G
TRPML3	RV	GAG ACC ATG TTC AGA GAAA CGA A

Upper lane: forward primer, lower lane: reverse primer.

### Cell proliferation

Cell proliferation was assessed using the CellTiter-Blue cell viability assay (Promega, Madison, WI, USA). Cells were treated as indicated for 72 h and 4 h before termination CellTiter-Blue reagent was added in a ratio of 1:10 (VCR-R CEM, CCRF-CEM, Jurkat) or 1:5 (HeLa). Fluorescence intensity was determined by using the SpectraFluor Plus plate reader (Tecan, Männedorf, Switzerland) at 550 nm excitation and 595 nm emission wavelength. Fluorescence intensity is proportional to cell number. Relative proliferation was calculated as follows:${{{\mathrm{relative}}}}\,{{{\mathrm{proliferation}}}} = \frac{{{{{\mathrm{x}}}} - {{{\mathrm{zero}}}}\,{{{\mathrm{value}}}}}}{{{{{\mathrm{control}}}} - {{{\mathrm{zero}}}}\,{{{\mathrm{value}}}}}}$. Non-linear regression was carried out using the log(inhibitor) vs. response–variable slope (four parameters) function of GraphPad Prism 8 (San Diego, USA).

### Colony formation

Colony formation experiments were conducted as described previously by Koczian et al. [26]. Colonies were stained with MTT (0.5 mg/ml for 2 h at 37 °C). Relative colony area was quantified using ImageJ by converting images to 8-bit, adjusting the threshold, and creating a selection to calculate the cell-covered area.

### Flow Cytometry

To determine apoptosis according to Nicoletti et al. [27], cells were treated as indicated, collected by centrifugation, and washed with ice-cold PBS. After resuspension in fluorochrome solution (50 µg/ml propidium iodide in deionized water containing 0.1% sodium citrate and 0.1% Triton-X 100), cells were incubated for 30 min at 4 °C. Cells with subG1 DNA content were considered apoptotic. Cell cycle analysis was performed accordingly and the G2 population was quantified. For PDX leukemia cells, cell death was determined by FSC/SSC, relying on cellular shrinkage and thus decrease in FSC upon cell death [28]. Cells were collected by centrifugation, washed with PBS, and resuspended in PBS. The respective cell population with decreased FSC intensity was considered dead. To determine lysosomal volume or pH, cells were either treated as indicated or left untreated and loaded with 200 nM LysoTracker Red or Green (Invitrogen, Waltham, MA, USA) at 4 °C or with 1 µM LysoSensor Green (Invitrogen) at 37 °C for 30 min and subsequently washed and resuspended in ice-cold PBS. To determine the influence of doxorubicin on lysosomal pH, cells were treated for 30 min with doxorubicin. To determine intracellular doxorubicin levels, cells were loaded with doxorubicin as indicated and subsequently washed and resuspended in ice-cold PBS. All flow cytometry experiments were performed on a BD FACS Canto II (BD Biosciences, Franklin Lakes, NJ, USA). Fluorescence intensity of propidium iodide, LysoTracker Red, and doxorubicin-loaded cells was analyzed using the PI channel, and fluorescence intensity of LysoSensor Green and LysoTracker green-loaded cells were analyzed using the FITC channel. Data were evaluated using FlowJo 7.6.

### Immunoblotting

Cells were either treated as indicated or left untreated, collected by centrifugation and washed twice with ice-cold PBS. For whole-cell lysates, cells were lysed in detergent-containing buffer (1% NP-40, 0.1% SDS, 0.25% deoxycholate, 150 mM NaCl, 50 mM Tris-HCl in deionized water; pH 7.5). Protease inhibitor Complete (Roche, Basel, Switzerland) was added directly before use. Subcellular fractionation was performed to isolate mitochondria according to the “mitochondrial purification protocol for western blot samples” from Abcam (Cambridge, UK). Isolation of lysosomes [29] was carried out as described previously. Protein content was analyzed by Bradford assay versus a BSA standard curve by measuring absorbance at 592 nm on a plate reader. Adequate amounts of 5x sample buffer (3.125 M Tris-HCl (pH 6.8), 50% glycerol, 5% SDS, 2% DTT, 0.025% Pyronin Y) and 1x sample buffer were added to adjust protein concentrations. Samples were subjected to SDS-PAGE (100 V for 21 min, 200 V for 40 min). Protein loading on gels was determined using stain-free technology and images were acquired on a Chemidoc imaging system (Bio-Rad Laboratories, Hercules, CA, USA). Proteins were transferred to PVDF membranes by tank blotting (100 V, 1.5 h, 4 °C). Membranes were washed with TBS-T and blocked with 5% BSA in TBS-T. Proteins were detected with specific primary antibodies, precisely PARP ((Cell Signaling Technology (CST), Frankfurt, Germany) #9542, 1:1000), active caspase 3 (Sigma C8487, 1:1000), γH2AX (CST #2577, 1:1000), LAMP1 (1:200, H4A3 Developmental Studies Hybridoma Bank), Hsp70 ((Santa Cruz Biotechnology, Dallas, TX, USA) sc-1060, 1:200), Bcl-xL (CST #2762), Bcl-2 (CST #2872), Bax (sc-493, 1:200), ATM (Sigma A1106, 1:2000), phospho-ATM Ser-1981 (CST #5883, 1:1000), p53 (CST #9282, 1:1000), phospho-p53 Ser-15 (CST #9284, 1:1000), Caspase 9 (CST #9502, 1:1000), Cathepsin B (CST #31718, 1:500), Bid (CST #2002, 1:1000), Vinculin (sc-25336, 1:500), VDAC (CST #4866, 1:1000), Cytochrome c (CST #4272, 1:1000), P-glycoprotein (Abcam, ab170904, 1:2000) and corresponding HRP-coupled secondary antibodies using a 2.5 mM luminol-containing solution. Membranes were imaged using a ChemiDoc Touch imaging system (Bio-Rad). Data were processed in ImageLab (Bio-Rad), and protein band intensities were normalized to protein amount on the gel (stain-free detection). Full and uncropped blots are supplied as supplementary data.

### Confocal microscopy

For confocal microscopy analysis of fixed cells, cells were immobilized on IbidiTreat µ-slides 8 well (ibidi, Graefelfing, Germany) by gravity sedimentation as described previously [30]. After treatment as indicated, cells were resuspended in PBS and incubated for 30 min at room temperature. For LysoTracker Red and LysoSensor Green experiments, PBS contained 200 nM LysoTracker Red or 1 µM LysoSensor Green, respectively. Subsequently, cells were fixed in 4% PFA and nuclei were counterstained with Hoechst 33342 (100 µg/ml). Samples were mounted in FluorSave Mounting medium, sealed with glass coverslips, and analyzed on a Leica TCS SP8 confocal microscope (Leica, Wetzlar, Germany) and Las X software (Leica).

Lysosomal leakage was analyzed with acridine orange adapted from previous descriptions [31, 32]. Cells loaded with 2 µg/ml acridine orange were exposed to high laser intensity (FRAP mode, 60% of maximal power of 488 nm laser) and pictures were taken after every exposition. Green and red fluorescence intensity of acidic vesicles were acquired in each picture. Lysosomal leakage leads to a loss in red and an increase in green fluorescence. Changes in red to green fluorescence intensity ratio were quantified at acidic spots. Brightness and contrast were adjusted in Fig. 4B, F, H and 5D to improve visibility. Original images are supplied as supplementary data.

### Cathepsin B activity

Cathepsin B activity was determined using the Cathepsin B Activity Assay Kit (PK-CA577-K140) (PromoKine, Heidelberg, Germany) according to the manufacturer’s instructions. Equal amounts of protein for each sample were used for each reaction. Cathepsin B-mediated cleavage of the substrate RR-AFC leads to a release of free AFC which is highly fluorescent. Fluorescence was measured on a Tecan plate reader (excitation wavelength: 390 nm, emission wavelength: 535 nm).

### Statistical analyses

Experiments were carried out at least three times independently unless stated otherwise. Data represent mean ± standard deviation (SD) unless stated otherwise. Statistical significance between two samples was determined by a two-tailed student’s *t*-test with Welch’s correction, if necessary unless stated otherwise. Statistical significance between more than two samples was determined by ordinary one-way ANOVA with Dunnett’s posttest. Statistical significance between two groups with different samples was determined with ordinary two-way ANOVA with Sidak’s or Tukey’s posttest. Results were considered significant for *p* < 0.05. Statistical tests were conducted in GraphPad Prism 8. The significance of differences in dose-response curves was analyzed using the comparison of fits function of GraphPad Prism 8, comparing logIC50 or logEC50 values resulting from the respective dose-response curve.

## Results

### Lysosomal role in chemo response and resistance

**Fig. 1:**
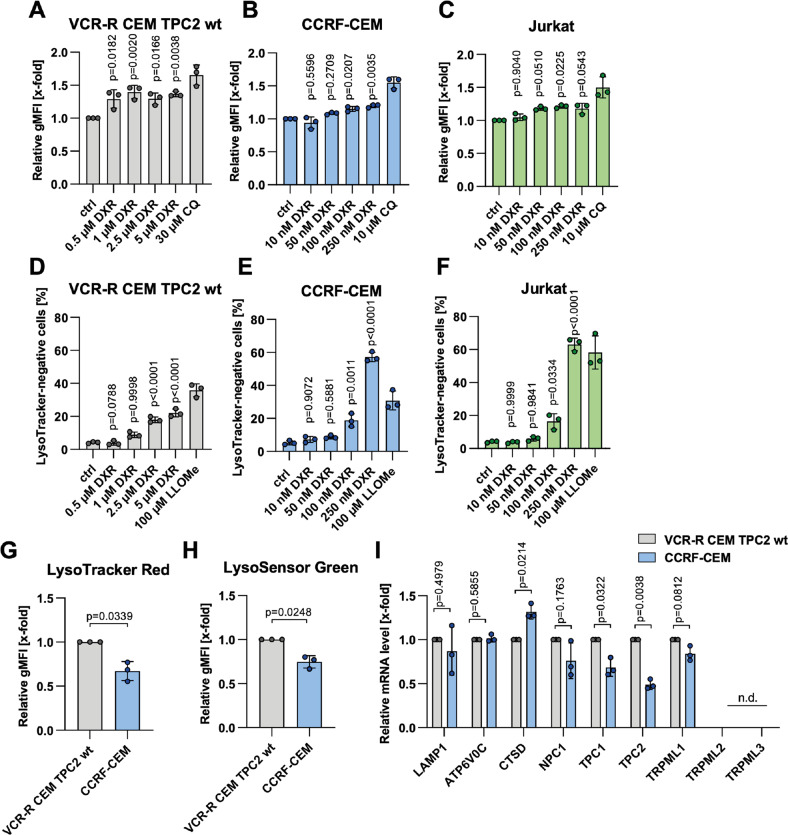
Role of lysosomes in chemo response and resistance. **A**–**C** Lysosomal volume was assessed by LysoTracker Green staining and flow cytometry after 4 h (VCR-R CEM TPC2 wt), **A** or 8 h (CCRF-CEM, **B** Jurkat, **C** of treatment). **D**–**F** Lysosomal damage was assessed by quantification of LysoTracker-negative cells by flow cytometry after 24 h of treatment. Chloroquine (CQ) **A**–**C** and L-Leucyl-L-Leucine methyl ester (LLOMe) **D**–**F** served as a positive control. **G** Lysosomal volume was assessed by LysoTracker Red staining and flow cytometry. **H** Lysosomal acidity was assessed by LysoSensor Green staining and flow cytometry. Cells with more acidic lysosomes show increased LysoSensor fluorescence. **I** The expression of lysosomal genes was analyzed by qPCR. Data were acquired from at least three independent experiments. Statistical significance was analyzed by one-way ANOVA with Dunnett’s post-test. **A**–**F** or student’s *t*-test with Welch’s correction **G**–**I**. Data are shown as mean ± SD.

Firstly, we evaluated the involvement of lysosomes in chemotherapy response in drug-naïve CCRF-CEM, drug-resistant VCR-R CEM (Fig. S1A), and Jurkat cells. By analyzing LysoTracker intensity after doxorubicin treatment, we found an increase in lysosomal volume, pointing to the induction of lysosomal stress. (Fig. 1A–C). This phenotype was also observed for topotecan, but not vincristine treatment in VCR-R CEM cells (Fig. S1B). Generally, induction of lysosomal stress may cause induction of lysosomal biogenesis. We found that initially lysosomal biogenesis is not induced, but can be detected with increasing exposition time, as indicated by the expression level of several markers (Fig. S1D, E). In addition, treatment with cytostatics led to lysosomal damage in all tested cell lines, pointing to an important role of lysosomes in chemotherapy response of ALL cells (Fig. 1D–F and S1C). This became further evident by comparing drug-naïve CCRF-CEM and drug-resistant VCR-R CEM cells regarding their lysosomal characteristics. Interestingly, drug-naïve CCRF-CEM cells had a significantly lower lysosomal volume as indicated by LysoTracker Red staining (Fig. 1G) and lysosomes were additionally less acidic compared to those of drug-resistant VCR-R CEM cells (Fig. 1H). Moreover, we detected alterations in expression levels of several lysosomal ion channels and genes associated with lysosomal function. Among these, an increase in TPC2 mRNA expression was most prominent in VCR-R CEM cells as compared to CCRF-CEM cells (Fig. 1I). As TPC2 is a promising anti-cancer target [17–19], which is upregulated in hard-to-treat, drug-resistant VCR-R CEM cells, we chose this cell line as a model to analyze the role of TPC2 in chemoresistance in ALL. Thus, we generated a TPC2 ko subline, using CRISPR/Cas9 technology (Fig. S2).

### Loss of TPC2 function sensitizes VCR-R CEM cells to cytostatics

**Fig. 2:**
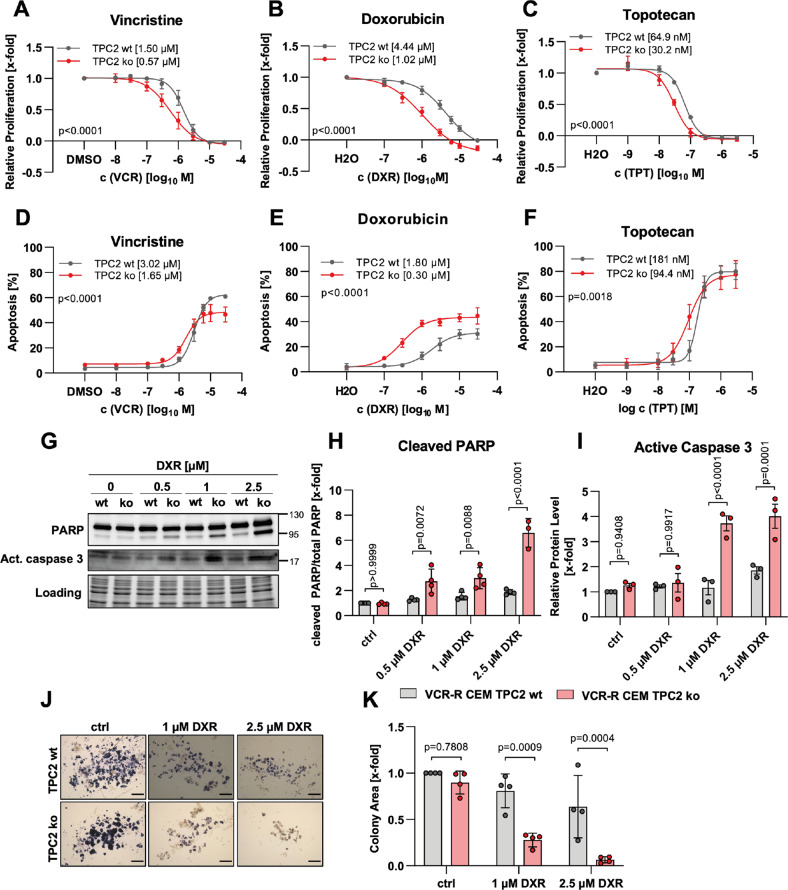
TPC2 knockout sensitizes VCR-R CEM cells to treatment with cytostatics. VCR-R CEM TPC2 wt and TPC2 ko cells were treated for 72 h (**A**–**C**) or 48 h (**D**–**F**). The antiproliferative effect of (**A**) vincristine, (**B**) doxorubicin, and (**C**) topotecan was assessed by CellTiter-Blue assay. Apoptosis induction after (**D**) vincristine, (**E**) doxorubicin and (**F**) topotecan treatment was assessed by propodium iodide staining and flow cytometry. **G** Immunoblots of VCR-R CEM wt and TPC2 ko cells. **H**, **I** Quantitative evaluation of protein levels from (**G**). **J** Cells were treated as indicated for 4 h and re-seeded in medium with 40% FCS and 0.4% methylcellulose and allowed to form colonies over 5 days of incubation. Living cells were stained with MTT reagent and images were analyzed in ImageJ (scale bar is 200 µm). Note that for evaluation color threshold of images was modified. **K** Quantitative evaluation of relative colony area of **J**. Data were acquired from at least three independent experiments. Statistical significance was analyzed using the comparison of fits function of GraphPad Prism 8, comparing logIC50 or logEC50 values (**A**–**F**) or two-way ANOVA with Sidak’s posttest (**H**, **I**, **K**). Data are shown as mean ± SD.

We then checked the sensitivity of TPC2 ko versus TPC2 wt cells to vincristine (VCR), doxorubicin (DXR), and topotecan (TPT). Our findings show that TPC2 ko sensitized VCR-R CEM cells to cytostatic treatment, evident by increased inhibition of proliferation and induction of apoptosis. Comparing IC50 and EC50 values showed that TPC2 ko cells are 2- to 4-fold more sensitive, depending on the cytostatic agent (Fig. 2A–F). This could also be verified by a significant increase of cleaved PARP and active caspase 3 in DXR-treated TPC2 ko cells as compared to TPC2 wt cells (Fig. 2G–I). Supporting these data, all cytostatics caused an increased cell cycle arrest in the G2/M phase in TPC2 ko cells (Fig. S3). Further, inhibition of colony formation by DXR treatment was more pronounced in TPC2 ko cells, as indicated by significantly decreased colony area (Fig. 2J, K). These data clearly point to an involvement of TPC2 in chemoresistance.

### Pharmacological TPC2 inhibition sensitizes leukemic cells to vincristine

**Fig. 3:**
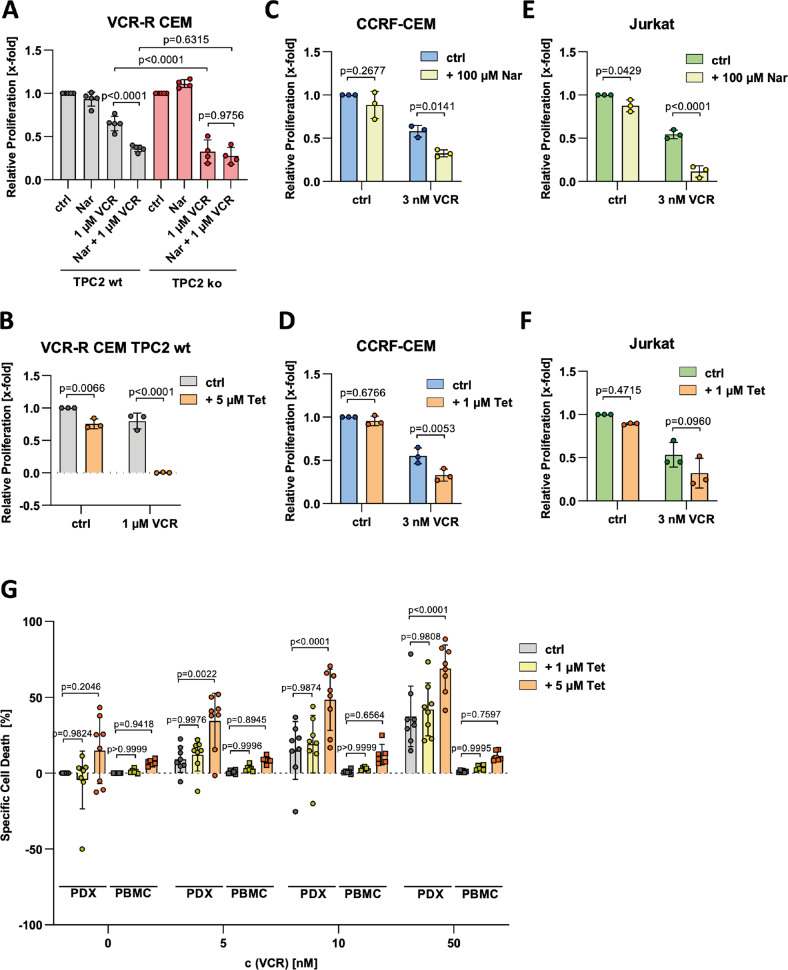
Pharmacological TPC2 inhibition sensitizes leukemic cells to vincristine. VCR-R CEM TPC2 wt (**A**, **B**) and TPC2 ko (**A**), CCRF-CEM (**C**, **D**), and Jurkat (**E**, **F**) cells were treated for 72 h and relative proliferation was assessed by CellTiter-Blue assay. **G** Isolated patient-derived xenograft cells (PDX, *n* = 8; circles) and peripheral blood mononuclear cells (PBMC, *n* = 6; squares) were treated for 48 h and cell death was analyzed employing FSC/SSC plot by flow cytometry. Data were acquired from at least three independent experiments. Statistical significance was analyzed by two-way ANOVA with Tukey’s posttest (**A**, **G**) or Sidak’s posttest (**B**–**F**). Data are shown as mean ± SD.

To confirm TPC2 as an appropriate target for chemosensitization and to elucidate druggability, we next employed pharmacological TPC2 inhibitors, namely naringenin and tetrandrine [19, 33]. Combining VCR with the citrus flavonoid naringenin leads to stronger cytotoxic effects in TPC2 wt cells as compared to vincristine single treatment. Importantly, combination with naringenin did not further sensitize TPC2 ko cells to treatment with vincristine, confirming an on-target effect of naringenin (Fig. 3A). Moreover, co-treatment with tetrandrine potentiated toxicity of vincristine (Fig. 3B), doxorubicin, and topotecan (Fig. S4A), whereas naringenin combined with doxorubicin or topotecan was not beneficial as compared to single treatment (Fig. S4B).

As VCR-R CEM cells express high levels of endogenous P-glycoprotein (P-gp) (Fig. S4C) due to the acquired resistance phenotype [21], we were interested in a potential connection between TPC2 and P-gp. Yet, we found that despite alterations in P-gp levels and intracellular distribution (Fig. S4D), P-gp is not responsible for the observed phenotype. This is obvious as treatment with cytostatics in combination with the P-gp inhibitors ketoconazole and verapamil revealed no alignment in response (Fig. S4E). Further, a combination of VCR with the small molecule TPC2 inhibitors naringenin and tetrandrine resulted in chemo sensitization also in the drug-naïve cell lines CCRF-CEM and Jurkat, suggesting a general chemosensitizing effect of TPC2 inhibition in leukemic cell lines, independent of P-gp expression and not restricted to resistant cells (Fig. 3C–F). In addition, HeLa and HepG2 cells were more sensitive to vincristine and doxorubicin when combined with naringenin or tetrandrine (Fig. S4F, G).

Patient-derived xenograft leukemia cells (PDX ALL, Fig. S4H) were utilized as an established in vitro model for heterogeneity in the clinic. Combining different VCR concentrations with 1 or 5 µM tetrandrine leads to significantly increased apoptosis as compared to monotherapy (Fig. 3F, also see Fig. S4I for detailed statistical analysis). These results underline, that targeting TPC2 in combination with cytostatic treatment is beneficial regarding induction of apoptosis also in PDX cells. At the same time, a combination of tetrandrine and vincristine has no significant impact on apoptosis in healthy peripheral blood mononuclear cells, suggesting a relative cancer specificity thereby providing a therapeutic margin of this approach (Fig. 3F).

### Loss of TPC2 function alters intracellular drug distribution

**Fig. 4:**
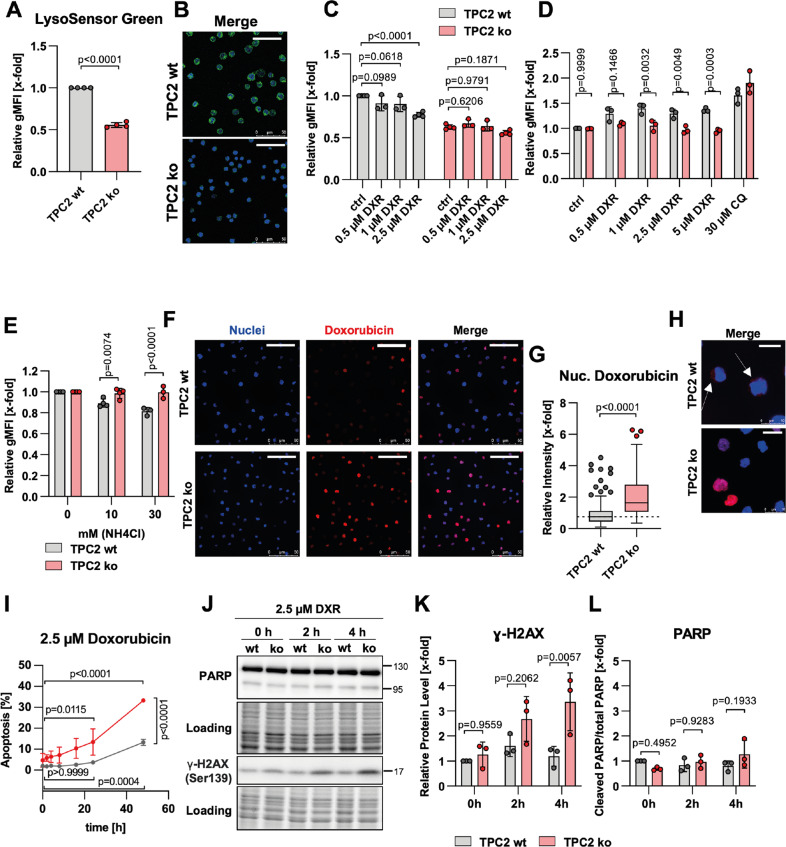
Loss of TPC2 function alters intracellular drug distribution. **A** Lysosomal acidity was assessed by LysoSensor Green staining and flow cytometry. Cells with more acidic lysosomes show increased LysoSensor fluorescence. **B** VCR-R CEM TPC2 wt and TPC2 ko cells were loaded with LysoSensor Green and fluorescence intensity was analyzed by confocal microscopy (scale bar is 50 µm). **C** Lysosomal pH was assessed by LysoSensor Green staining and flow cytometry after 30 min of treatment. **D** Lysosomal volume was assessed by LysoTracker Green staining and flow cytometry after 4 h of treatment. **E** VCR-R CEM TPC2 wt and TPC2 ko cells were treated with 5 µM doxorubicin in the presence or absence of different concentrations of the lysosomotropic compound ammonium chloride for 24 h. Intracellular doxorubicin fluorescence was analyzed by flow cytometry. **F** VCR-R CEM TPC2 wt and TPC2 ko cells were treated for 4 h and nuclear doxorubicin (red) was determined by co-staining with Hoechst 33342 (blue) and confocal microscopy (scale bar is 50 µm) and quantified (**G**) using ImageJ. 119 TPC2 wt and TPC2 ko cells were analyzed in three independent experiments and data points are displayed as box and whiskers with Tukey depiction. **H** Zoom from (**F**). Arrows point to the cytoplasmic abundance of doxorubicin (scale bar is 10 µm). Note that brightness and contrast were adjusted to improve visibility (**B**, **F**, **H**). **I** VCR-R CEM TPC2 wt and TPC2 ko cells were treated with 2.5 µM doxorubicin, and apoptosis was analyzed by propidium iodide staining and flow cytometry. **J** Immunoblots of VCR-R CEM wt and TPC2 ko cells. **K**, **L** Quantitative evaluation of protein levels from (**J**). Data were acquired from at least three independent experiments. Statistical significance was analyzed by student’s *t*-test with Welch’s correction (**A**), two-way ANOVA with Sidak’s posttest (**C**, **D**, **E**, **K**, **L**), Mann–Whitney test (**G**), or two-way ANOVA with Tukey’s posttest (**I**). Data are shown as mean ± SD, apart from (**G**).

Sequestration of cytostatic drugs within lysosomes and subsequent prevention from reaching their intracellular targets has been reported as an important mechanism of chemoresistance [10, 11]. As lysosomal pH is an important regulator on this occasion, we performed semi-quantitative pH measurements. These exhibited that lysosomes of TPC2 ko cells were less acidic, indicated by loss of LysoSensor fluorescence intensity (Fig. 4A, B). Hence, we compared lysosomal sequestration of doxorubicin in TPC2 wt and TPC2 ko cells. Upon lysosomal sequestration of weak bases, lysosomal pH is increased. LysoSensor staining showed increased lysosomal pH and thus sequestration of DXR only in TPC2 wt cells, but not in TPC2 ko cells at any tested concentration (Fig. 4C). Along the line, increased lysosomal volume as a result of lysosomal stress could only be detected in TPC2 wt (Fig. 4D). Of note, similar results were observed in topotecan-treated cells (Fig. S5A). In addition, a combination of DXR with the lysosomotropic compound ammonium chloride causes a decrease in intracellular DXR levels in TPC2 wt cells, but not in TPC2 ko cells (Fig. 4E), emphasizing differences in DXR sequestration capability. Underlining the involvement of lysosomal drug sequestration in TPC2 ko-mediated chemo sensitization, there were no differences in sensitivity to non-basic cytostatics observable (Fig. S5B).

Consequently, TPC2 ko cells showed significantly more DXR in nuclear regions as compared to TPC2 wt cells after 4 h of treatment (Fig. 4F, G). Further, doxorubicin fluorescence was almost exclusively found in nuclear regions in TPC2 ko cells, while TPC2 wt cells additionally had extra-nuclear abundance (Fig. 4H, arrows). As it is a DNA intercalating agent, increased nuclear presence of DXR should increase drug-induced DNA damage and cell death. To verify this hypothesis, it is necessary to exclusively analyze doxorubicin-induced DNA damage instead of apoptosis-induced DNA fragmentation. Time-dependent assessment of apoptosis revealed apoptosis induction after 24 h, which was more prominent in TPC2 ko cells than in TPC2 wt cells (Fig. 4I). Quantification of DNA damage by γH2AX detection revealed increased DNA damage in TPC2 ko cells already after 4 h of treatment, whereas significant PARP cleavage was not yet detectable at this time point (Fig. 4J–L). Since TPC2-mediated calcium flux is an important regulator of TFEB, which was previously implicated in p53-mediated DNA damage response [34], we additionally investigated the functionality of the DNA-damage response machinery. However, no alterations in repair-associated proteins p53 and ATM could be observed (Fig. S5C). In summary, drug sequestration is impaired in TPC2 ko cells, leading to increased nuclear doxorubicin, and subsequently increased DNA damage induction, while DNA repair itself seems not to be affected by a loss of TPC2 function.

### TPC2 ko cells are more susceptible to lysosomal damage

**Fig. 5:**
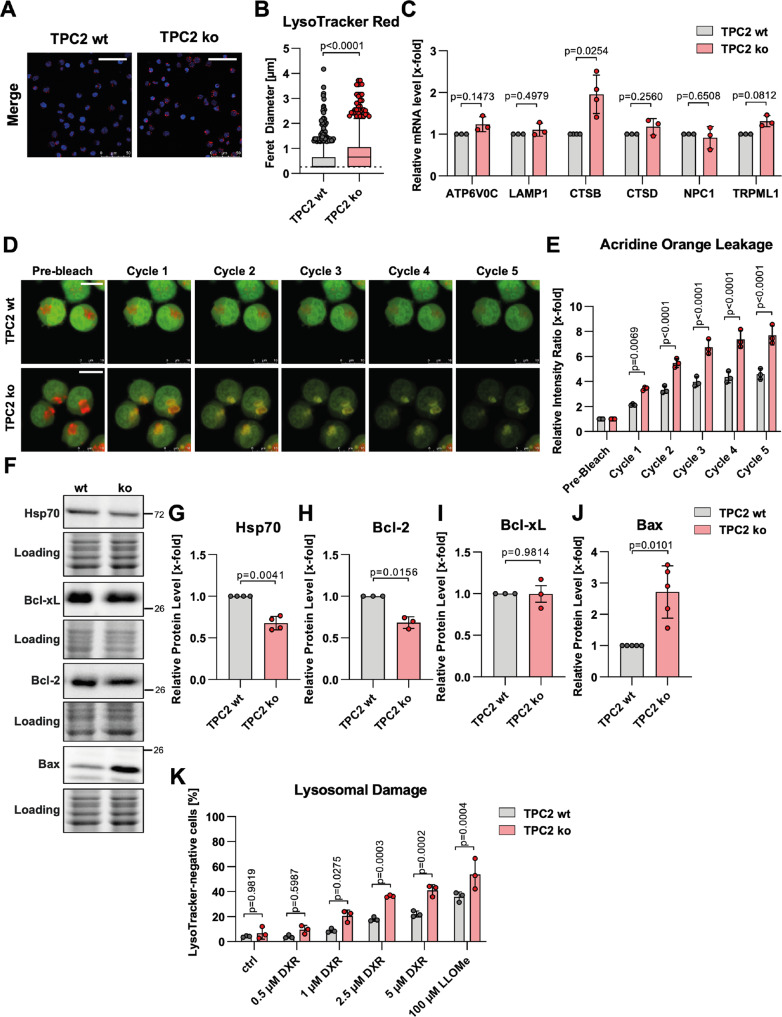
Lysosomal dysfunction in TPC2-deficient cells promotes lysosomal membrane permeabilization. **A** Lysosomes of untreated VCR-R CEM TPC2 wt and ko cells were stained with LysoTracker Red (red), counterstained with Hoechst 33342 (nuclei; blue), and analyzed by confocal microscopy (scale bar is 50 µm). **B** Quantitative evaluation of Feret diameter of lysosomes from **A**. The lysosomal diameter was determined using ImageJ. 2684 lysosomes of TPC2 wt and 1644 lysosomes of TPC2 ko cells were analyzed in three independent experiments and data points are displayed as box and whiskers with Tukey depiction. **C** The expression of lysosomal genes was analyzed by qPCR. **D** VCR-R CEM TPC2 wt and TPC2 ko cells were loaded with acridine orange and analyzed by confocal microscopy before or after repeated exposition to high-intensity laser light. After each cycle of exposition images were taken (scale bar is 10 µm). Note that brightness and contrast were adjusted to improve visibility. **E** Quantitative evaluation of **D**. Green and red fluorescence intensity values were analyzed at acidic spots (= high red fluorescence intensity) using ImageJ. For evaluation, the ratio of green to red fluorescence intensity was calculated. **F** Immunoblots of untreated VCR-R CEM TPC2 wt and TPC2 ko cells. **G**–**J** Quantitative evaluation of protein levels from **F**. **K** Lysosomal damage was analyzed by LysoTracker Green staining and flow cytometry. LysoTracker-negative cell population was considered as a population with damaged lysosomes. LLOMe served as a positive control for lysosomal damage induction. Data were acquired from at least three independent experiments. Statistical significance was analyzed by Mann–Whitney test (**B**), student’s *t*-test with Welch’s correction (**C**, **G**–**K**), or two-way ANOVA with Sidak’s posttest (**E**). Data are shown as mean ± SD, apart from (**B**).

Evaluating further lysosomal characteristics, confocal microscopy revealed that lysosomes of TPC2 ko cells displayed a significantly enlarged diameter (Fig. 5A, B). Despite different morphology, expression levels of lysosome-associated genes were mostly unchanged, except for an increased expression of TRPML1 and CTSB in TPC2 ko cells (Fig. 5C). These findings suggest that lysosomal biogenesis, in general, is not substantially altered, yet lysosomes of TPC2 ko cells are swollen. Interestingly, increased lysosomal size has previously been connected with lysosomal stability and subsequently lysosomal cell death induction [9, 35]. Therefore, we used acridine orange, which accumulates in acidic compartments and shifts in emission wavelength from red to green upon laser-induced lysosomal damage. Interestingly, laser exposition leads to a stronger increase in green to red fluorescence ratio in TPC2 ko cells, indicative of increased induction of lysosomal membrane permeabilization and damage (Fig. 5D, E). Additionally, proteins connected to lysosomal stability [36] were differentially expressed in TPC2 ko cells. While the lysosome stabilizing protein Bcl-xL was not deregulated, Hsp70 and Bcl-2 were downregulated in TPC2 ko cells. Furthermore, the pro-apoptotic protein Bax, which is thought to be lysosome-destabilizing, was upregulated (Fig. 5F–J).

Along the line, DXR-induced lysosomal damage was analyzed. TPC2 ko samples had a significantly larger population of LysoTracker-negative cells after DXR treatment than TPC2 wt cells. The same phenotype was observed with the widely used lysosomal damage inducer LLOMe (Fig. 5K). This also applied to vincristine and topotecan (Fig. S6). These results emphasize that loss of TPC2 function causes increased susceptibility to lysosomal damage, induced by either phototoxicity or cytostatics.

### Contribution of lysosomal damage to cell death

**Fig. 6:**
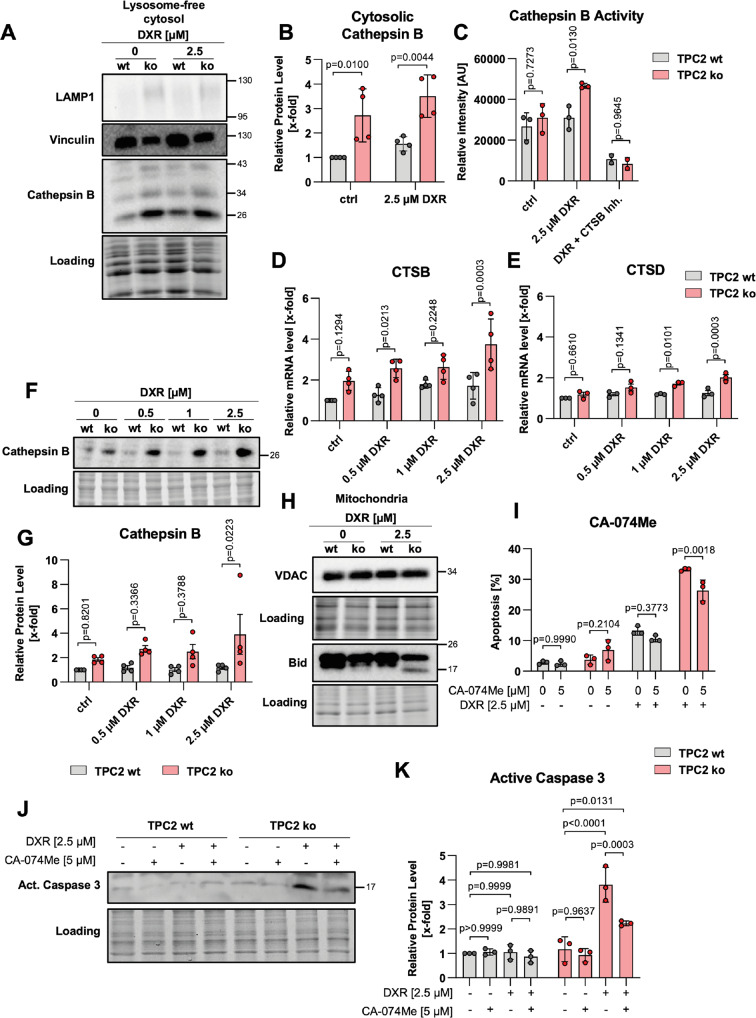
Lysosomal leakage and cathepsins are involved in the increased sensitivity of TPC2 knockout cells. **A** Immunoblots of lysates containing lysosome-free cytosol of VCR-R CEM TPC2 wt and TPC2 ko cells. **B** Quantitative evaluation of protein levels from (**A**). **C** Cathepsin B activity of VCR-R CEM TPC2 wt and TPC2 ko cells was determined in cell lysates using the substrate Ac-RR-AFC by quantifying fluorescence intensity of free AFC. **D**, **E** Expression levels of cathepsin B (**D**) and cathepsin D (**E**) of VCR-R CEM TPC2 wt and TPC2 ko cells were analyzed by qPCR. **F** Immunoblots of VCR-R CEM wt and TPC2 ko cells. **G** Quantitative evaluation of protein levels from (**F**). **H** Immunoblots of isolated mitochondria of VCR-R CEM TPC2 wt and TPC2 ko cells. **I** Apoptosis of VCR-R CEM TPC2 wt and TPC2 ko cells was determined by propidium iodide staining and flow cytometry. **J** Immunoblots of VCR-R CEM TPC2 wt and TPC2 ko cells. **K** Quantitative evaluation of protein levels from (**F**). Data were acquired from at least three independent experiments. Statistical significance was analyzed by two-way ANOVA with Sidak’s posttest (**B**–**E**, **G**, **K**) or Tukey’s posttest (**I**). Data are shown as mean ± SD.

As lysosomal damage can initiate LCD, we analyzed major LCD hallmarks upon DXR treatment. Cytosolic cathepsin B was analyzed after the separation of lysosomes, showing a higher cytosolic abundance in untreated and DXR-treated TPC2 ko cells as compared to TPC2 wt cells. (Fig. 6A, B). In addition, a fluorescence-based enzyme activity assay revealed increased cathepsin B activity in TPC2 ko cells upon DXR treatment (Fig. 6C). Furthermore, the expression of cathepsins D and B were found to be increased on mRNA (Fig. 6D, E) and protein level (Fig. 6F, G) in TPC2 ko cells, when treated with DXR. In line with that, truncated Bid, which is a main target of cathepsins and an important pro-apoptotic regulator, was detectable in mitochondrial fractions of TPC2 ko cells treated with DXR, whereas it was not detectable in treated TPC2 wt cells (Fig. 6H). Along the line, apoptosis of DXR-treated TPC2 ko cells was partially reversed when combined with the cathepsin B inhibitor CA-074Me (Fig. 6I). Further, inhibiting cathepsin B prevented DXR-induced caspase 3 activation in TPC2 ko cells (Fig. 6J, K). These findings suggest induction of cathepsin B-dependent LCD in TPC2 ko cells upon DXR treatment.

As LCD is known to crosstalk with mitochondria-mediated apoptosis and we observed Bid truncation, we analyzed the mitochondrial apoptosis pathway. Yet, we found that the mitochondria-mediated pathway does not play a major role in the DXR-induced cell death of drug-resistant VCR-R CEM cells. While we detected increased mitochondrial mass and reduced Hsp70 protein levels in isolated mitochondria, mitochondrial membrane potential was not depleted in TPC2 ko cells (Fig. S7A–C). Further, neither TPC2 wt nor TPC2 ko cells lost their mitochondrial integrity upon DXR treatment, as no cytochrome c release or activating cleavage of caspase 9 was detectable (Fig. S7D, E). These data emphasize that TPC2 ko cells show an increased abundance of cytosolic cathepsin B and increased cathepsin B activity contributing to cell death, occurring independently from the intrinsic mitochondria-mediated apoptotic signaling pathway.

## Discussion

**Fig. 7:**
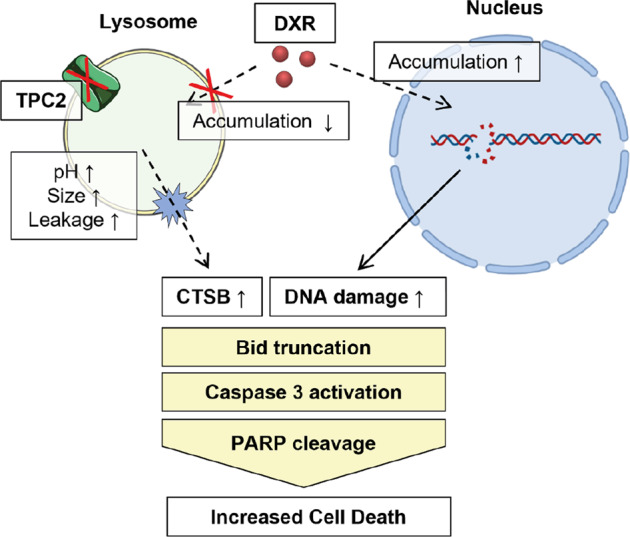
Proposed mechanism of chemo sensitization by loss of TPC2 function. Loss of TPC2 function causes impairment of lysosomal functionality, including increased pH, increased lysosomal size, and increased susceptibility to lysosomal damage. Hence, TPC2 ko cells are not able to sequester weak base drugs within lysosomes, causing accumulation at their target sites and thus increased effectivity. Concurrently, lysosomal damage initiates lysosomal cell death mediated by cytosolic cathepsin B. These two pathways increase pro-apoptotic signaling, e.g. activating truncation of Bid, activation of the executioner caspase 3 and PARP cleavage, ultimately causing increased cell death in TPC2 ko cells.

The work presented here establishes TPC2 as a promising target for combination therapy to improve response to cytostatics and overcome chemoresistance in leukemia. To date, chemoresistance still remains a therapy-limiting challenge in cancer treatment in which lysosomal function emerged as an interesting regulator. We now present evidence that TPC2 serves as a novel, druggable target to overcome this therapeutic drawback. Most importantly, we reveal that TPC2 ko or pharmacological inhibition sensitizes leukemia cells to treatment with widely used cytostatics like vincristine and doxorubicin. Mechanistically, chemo sensitization is achieved by loss-of-TPC2-function-induced alterations in lysosomal size and pH, hindering lysosomal drug sequestration and favoring lysosomal membrane damage, ultimately increasing cell death (Fig. 7).

The influence of TPC2 modulation on lysosomal function and characteristics is of high interest in recent research and many aspects such as lysosomal pH alterations are discussed controversially. Most available data originate from studies focusing on healthy, yet data on cancer cells are scarce. In skeletal muscle cells, loss of TPC2 function led to lysosomal alkalization [16] and acidity of melanosomes, a different type of acidic vesicles, depends on TPC2 function [37], which is in line with our data. In turn, Grimm et al. report no differences in lysosomal pH in TPC2 ko mouse embryonic fibroblasts [38] and another study reports that lysosomal pH was not changed upon activation of TPC2 with specific small molecules [14]. Hence, the exact role of TPC2 in vesicular pH regulation remains unclear and appears to be highly cell type-dependent. We are now able to provide additional data on this controversy, as our study reveals for the first time that lysosomal acidity can be manipulated by targeting TPC2 in leukemia cells, showing that ko of TPC2 leads to increased lysosomal pH. Besides the controversial findings on TPC2-dependent pH regulation, further lysosomal characteristics are reported to be affected by TPC2. Swollen lysosomes have been identified by Nguyen et al. in TPC2-depleted HUH7 and T24 cancer cells and upon pharmacological TPC2 inhibition, causing impaired integrin trafficking [19]. These data are in line with our observation of increased lysosomal diameter in TPC2-depleted cells.

It seems clear in the literature, that the functionality of lysosomes is critical for sequestration capability and capacity of weak basic compounds. Recently, inhibition of lysosomal drug sequestration has emerged as a promising approach to improve response to chemotherapy in pre-clinical studies [10]. Hitherto most available studies focus on combining anti-cancer agents with lysosomotropic compounds, a class of compounds accumulating inside the lysosomal lumen and thereby raising intraluminal pH. For example, chloroquine, ammonium chloride, and methylamine have been combined with doxorubicin, daunorubicin, and vinblastine, with the result that this combination is highly beneficial in chemo-resistant KBV1 cells [39]. Mechanistically, competition for lysosomal accumulation decreases sequestration of the anti-cancer agent [10]. Yet, target-specific and thus probably more selective approaches are scarce or face druggability problems. A study dealing with targeting the lysosomal V-ATPase used siRNA-mediated knockdown of its subunit ATP6L, which led to sensitization of doxorubicin-resistant MCF-7 cells to various cytostatics [40]. However, the clinical application of common V-ATPase inhibitors is limited by severe toxicity [41, 42]. Furthermore, V-ATPase inhibition with bafilomycin A1 did not have a beneficial effect on cytarabine sensitivity in chemo-resistant acute myeloid leukemia [43].

Our study now establishes TPC2 as a druggable target to overcome lysosomal drug sequestration and subsequent chemosensitization. Of note, recently published small molecule inhibitors and activators for TPC2 should allow modulation of channel activity in vivo [14, 17]. Besides establishing TPC2 as a novel target, we further provide novel mechanistic insights indicating a dual-mode of action. On the one hand, TPC2 loss of function impairs drug sequestration leading subsequently to a higher abundance of cytostatics at the target site and thus to an increased response to therapy. On the other hand, it significantly destabilizes lysosomes favoring lysosomal damage, evident by decreased stability of acridine orange-loaded acidic vesicles upon exposition to high-intensity laser light and quantification of lysotracker-negative cells by flow cytometry upon treatment with doxorubicin. Besides the induction of LCD, lysosomal damage, in turn, might contribute to impaired drug sequestration, as it causes a release of trapped cytostatics back into the cytosol. Hence, we propose that targeting the TPC2 function acts in a dual-mode of action and creates a feed-forward cycle resulting in chemosensitization of drug-naïve and as well as resistant cells.

Additionally, our study provides insight into LCD signaling upon TPC2 loss of function. Mechanistically, it has been shown that lysosomal damage leads to the release of lysosome-abundant proteases, most prominently cathepsins, capable of initiating and executing cellular death either caspase-dependent or caspase-independent [9, 44, 45]. Consequently, we elucidated the mode of LCD induction in our model and focussed on the impact of cathepsin B on cell death of TPC2 wt and TPC2 ko cells. Increased cathepsin B abundance in the cytosol of TPC2 ko cells, as well as increased activity and expression upon doxorubicin treatment, pointed to an important role of cathepsins, confirmed by reversion of cell death by combination with the cathepsin B inhibitor CA-074Me. Interestingly, cathepsin B-dependent apoptosis has also been related to increased lysosomal damage upon depletion of lysosomal cation channel TRPML1, underlining the importance of lysosomal cation channels for lysosomal function and homeostasis [46]. Given that lysosomes of cancer cells are more prone to lysosomal damage and thus provide a certain degree of cancer selectivity, provoking lysosomal damage to induce cell death gained huge interest [47–49]. For example, the acid sphingomyelinase inhibitors siramesine, desipramine and amlodipine effectively induce lysosomal damage and subsequent cell death in single treatment as well as in combination with paclitaxel [49]. Moreover, verteporfin, identified as a lysosome damaging compound, synergistically induces apoptosis when combined with sorafenib in hepatocellular carcinoma [50]. With our data, we can support these findings and additionally report for the first time that depletion of lysosomal cation channel TPC2 increases susceptibility to lysosomal damage. Our results indicate that the cytostatic agent doxorubicin induces lysosomal damage, which is in line with published data [47], and that this phenomenon is more prominent in TPC2 ko cells as compared to TPC2 wt cells.

LCD initiated by lysosomal damage is in extensive cross-talk with different apoptotic pathways. For instance, cytosol-abundant, active cathepsins degrade anti-apoptotic and activate pro-apoptotic proteins from the Bcl-2 family, favoring mitochondrial damage and subsequently intrinsic apoptosis [51, 52]. Yet, in our cell model, we could only detect minor alterations in the classical mitochondria-related pathway, such as truncation of pro-apoptotic Bid, alterations in mitochondrial mass, and decreased Hsp70 levels in mitochondria of TPC2 ko cells. However, mitochondrial outer membrane permeabilization does not occur, as indicated by the absence of cytosolic cytochrome c upon treatment with toxic doxorubicin concentrations. Of note, cytostatic-induced lysosomal damage might be related to the generation of reactive oxygen species reported to destabilize the lysosomal membrane and therefore likely to contribute to the phenotype [36, 53]. Our data emphasize that mitochondria-initiated apoptosis does not play a central role suggesting a main role of the endolysosomal system in initiating and executing cell death in chemo-resistant leukemic cells.

As we provide evidence, that lysosomal TPC2 represents a promising druggable target for a dual-mode of action strategy for combination therapies with cytostatics, we tested clinical relevance. A comparison of data obtained from healthy PBMC and heterogenous PDX ALL cells indicates cancer selectivity of TPC2 inhibition, in combination with vincristine as well as in monotherapy. These observations are in accordance with previous findings, which showed that TPC2 inhibition preferentially targets tumor cells. In addition, several studies have proven the druggability of TPC2 and identified different derivatives of tetrandrine and naringenin as novel pharmacological inhibitors with improved potency and drug-like properties [17, 18].

Summed up, we propose lysosomal TPC2 as a novel, effective and druggable target to circumvent chemoresistance caused by lysosomal drug sequestration. The study presented here provides evidence that TPC2 acts with a dual-mode of action. Loss of TPC2 function in our model impairs drug sequestration, increases drug presence at cellular targets, and induces lysosomal damage favoring cathepsin B-induced cell death.

## Supplementary information

Figure S1

Figure S2

Figure S3

Figure S4

Figure S4 continued

Figure S5

Figure S6

Figure S7

Supplementary Figure Legends

Supplementary Table

Full Blots and Original Images

AI Checklist

## Data Availability

All data generated or analyzed during this study are included in this published article and its supplementary information files. Any additional information related to data might be obtained from the corresponding author upon reasonable request.
