# Supplementary figures and images for: Targeting TPC2 sensitizes acute lymphoblastic leukemia cells to chemotherapeutics by impairing lysosomal function
Source: Cell Death Dis. 2022 Aug 1;13(8):668. doi: 10.1038/s41419-022-05105-z (PMC9343397; doi:10.1038/s41419-022-05105-z)

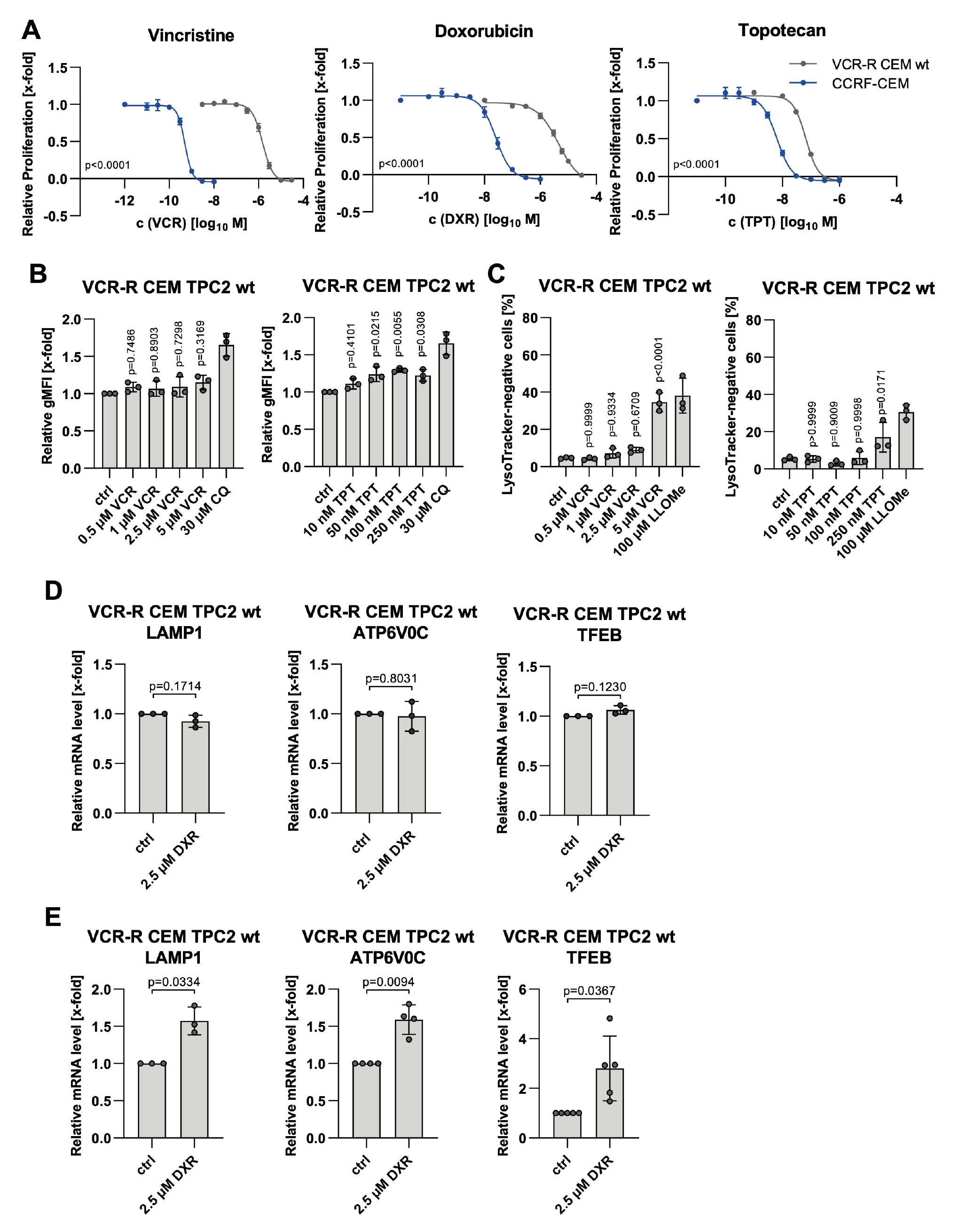

Supplement: Supplementary file 1 — Figure S1 [file 41419_2022_5105_MOESM1_ESM.png]

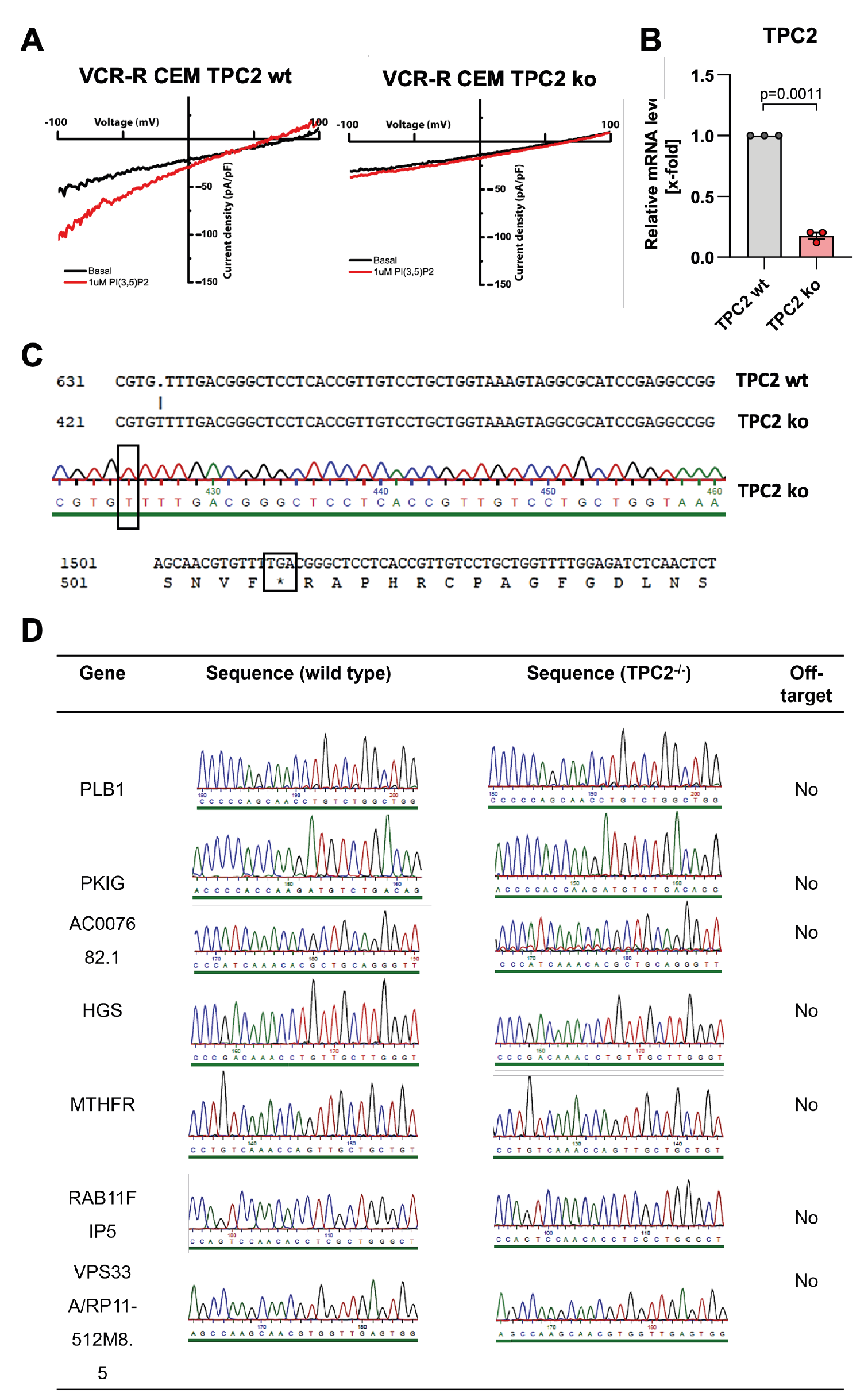

Supplement: Supplementary file 2 — Figure S2 [file 41419_2022_5105_MOESM2_ESM.png]

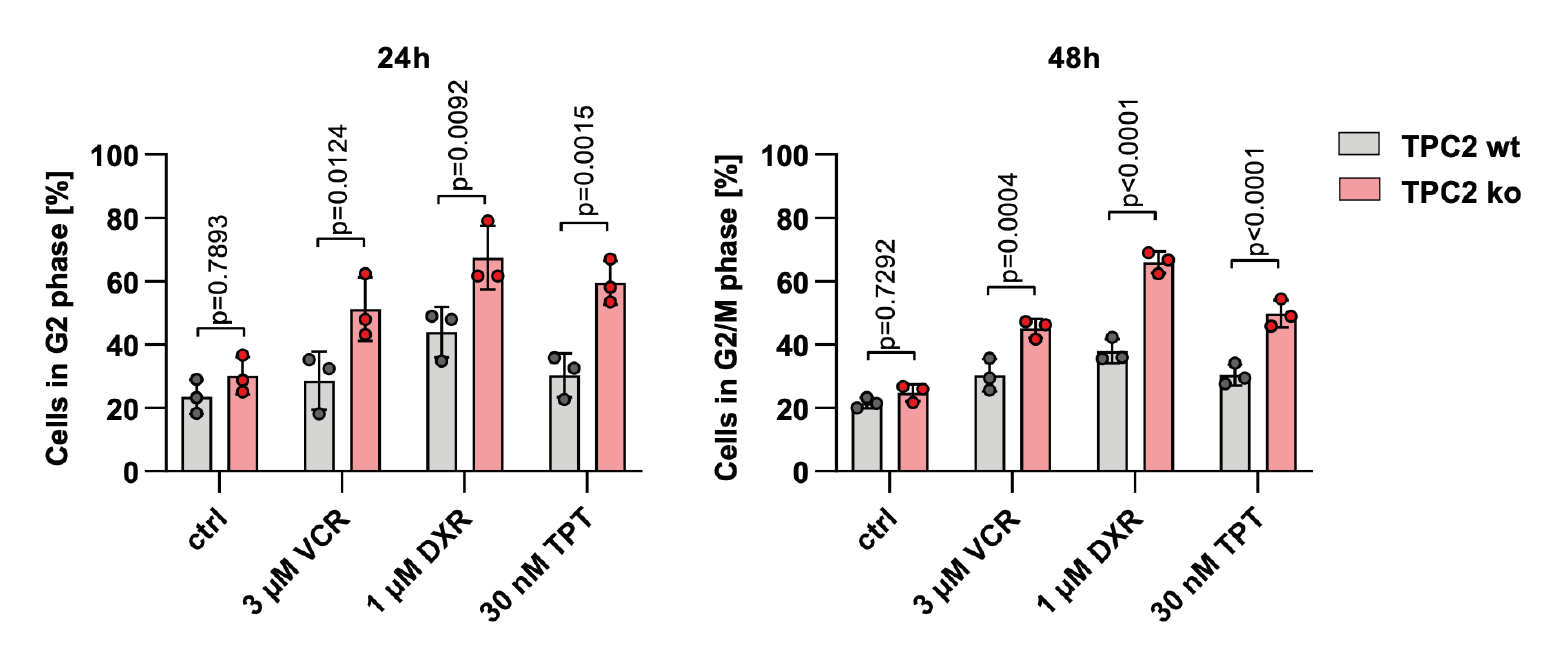

Supplement: Supplementary file 3 — Figure S3 [file 41419_2022_5105_MOESM3_ESM.png]

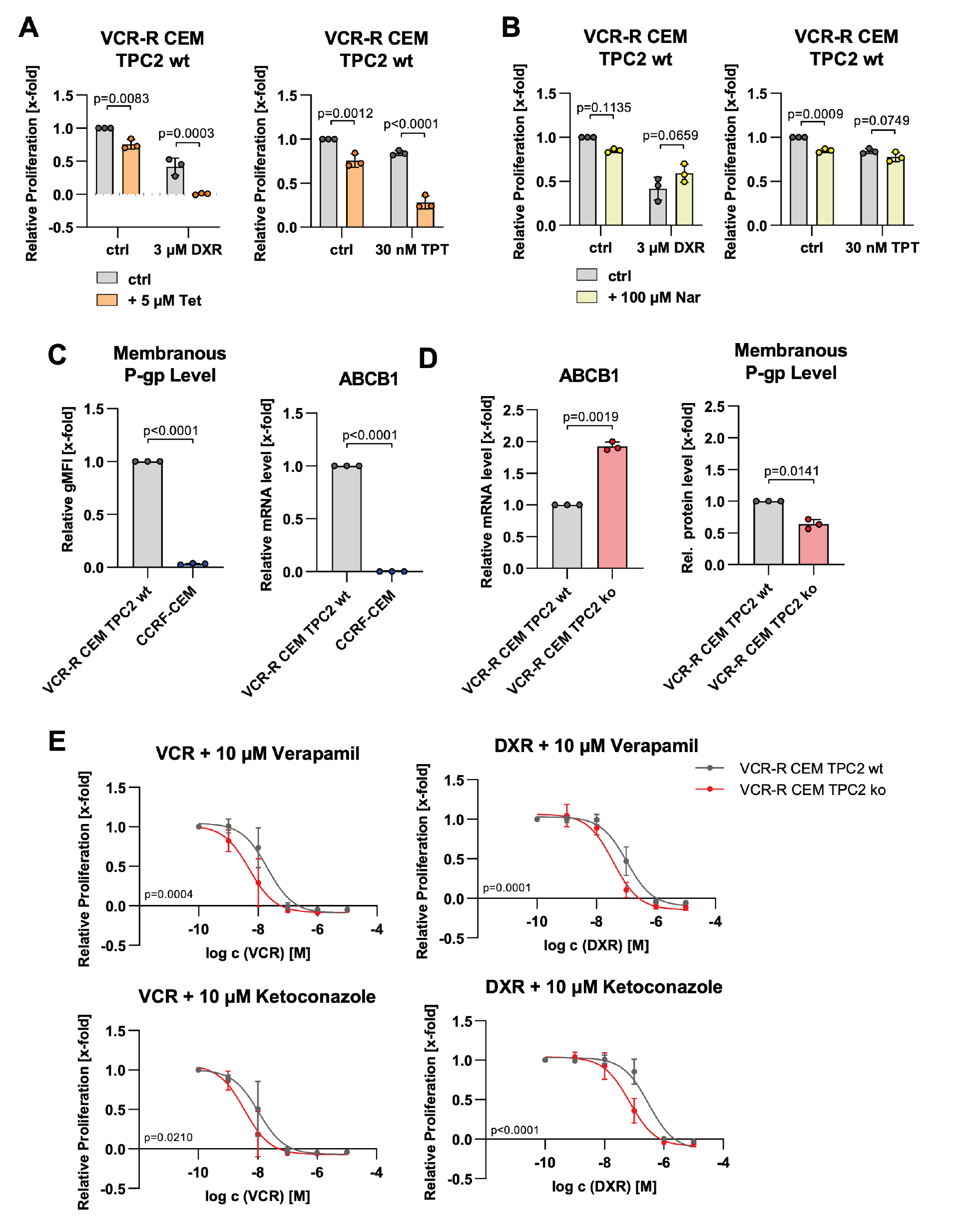

Supplement: Supplementary file 4 — Figure S4 [file 41419_2022_5105_MOESM4_ESM.png]

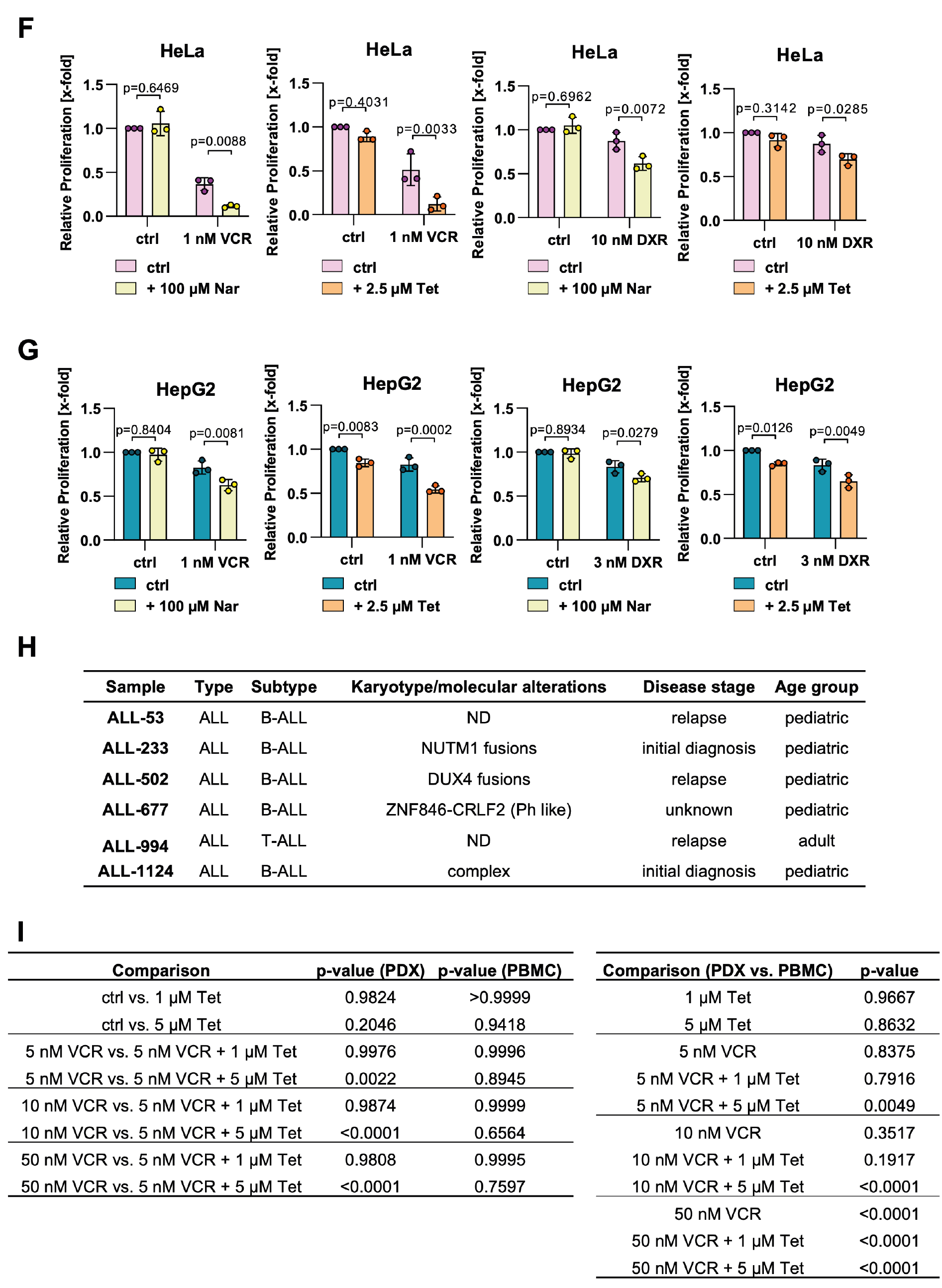

Supplement: Supplementary file 5 — Figure S4 continued [file 41419_2022_5105_MOESM5_ESM.png]

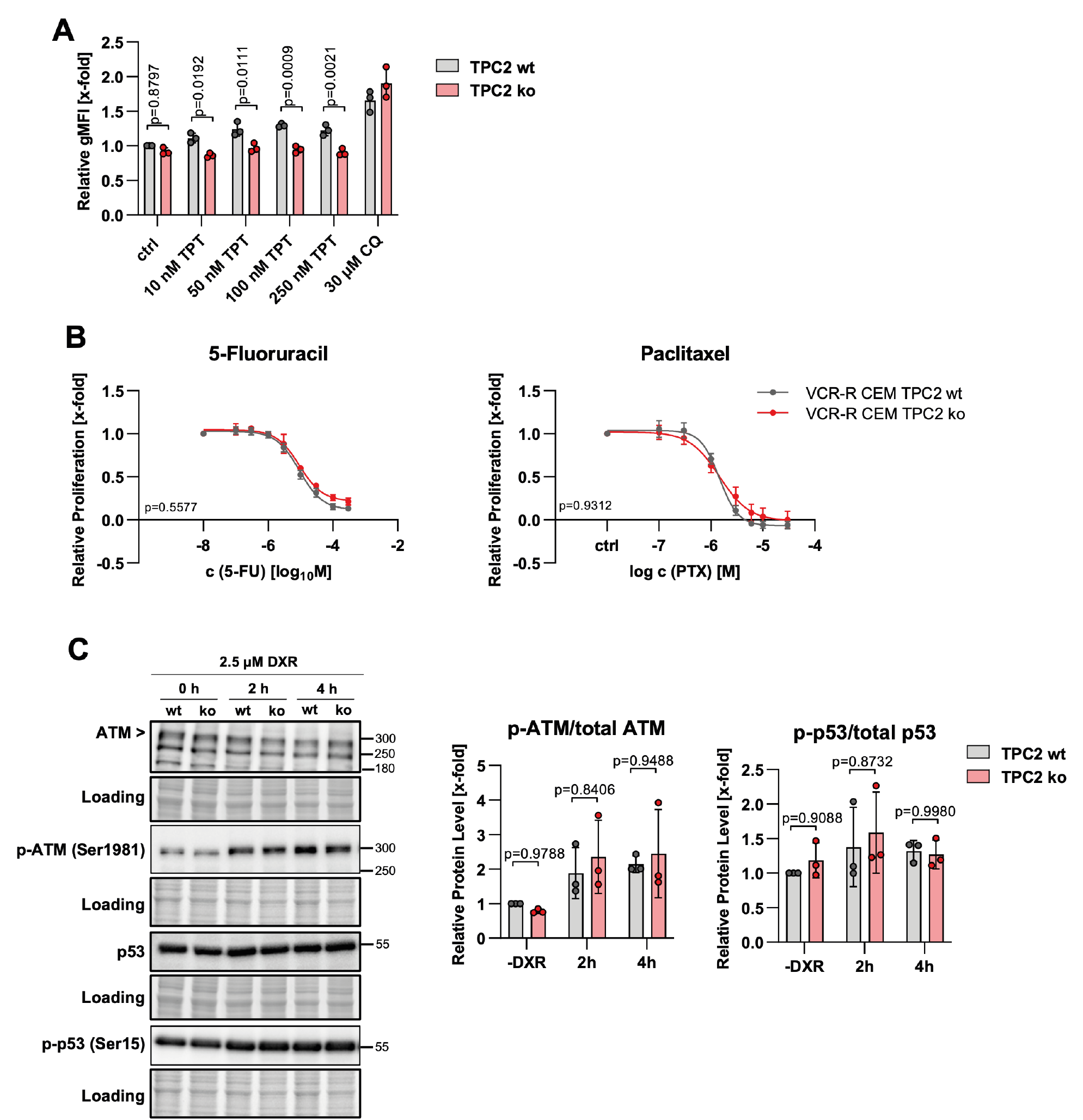

Supplement: Supplementary file 6 — Figure S5 [file 41419_2022_5105_MOESM6_ESM.png]

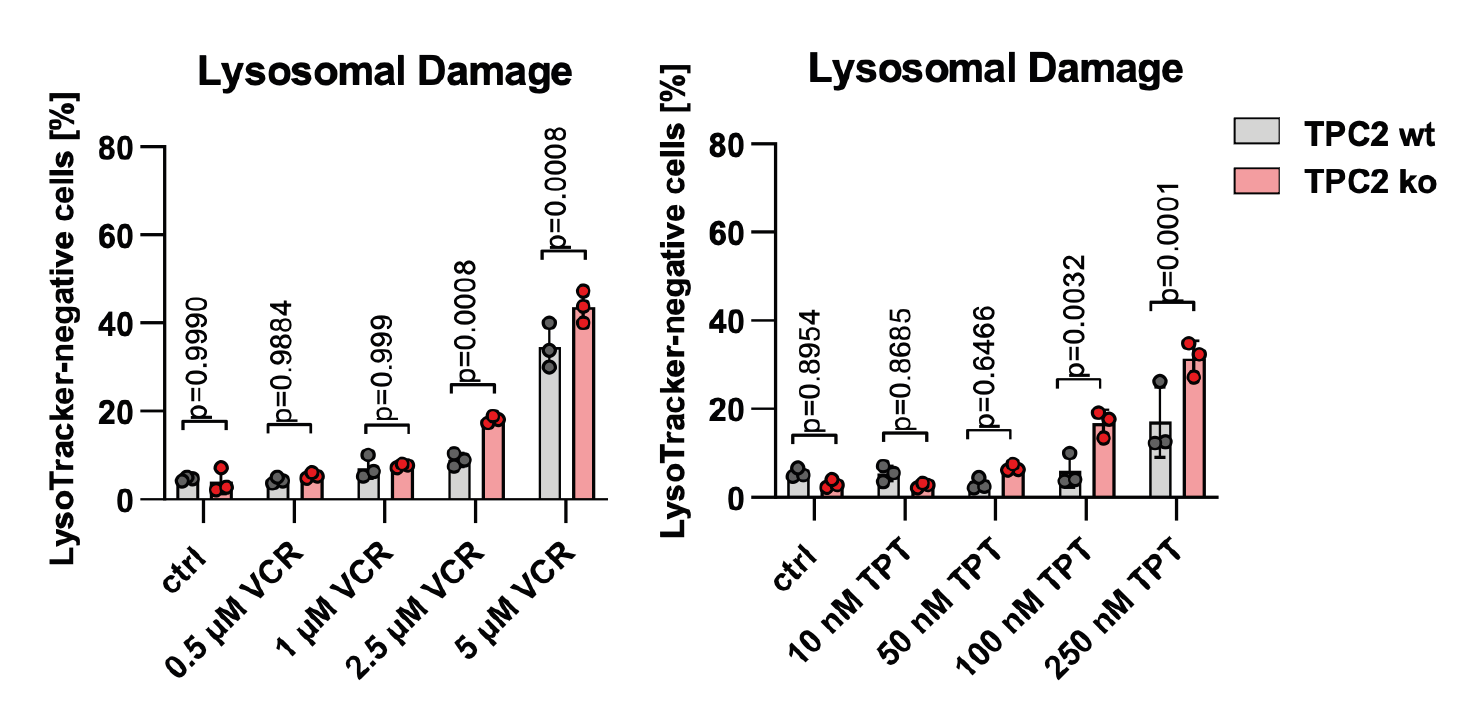

Supplement: Supplementary file 7 — Figure S6 [file 41419_2022_5105_MOESM7_ESM.png]

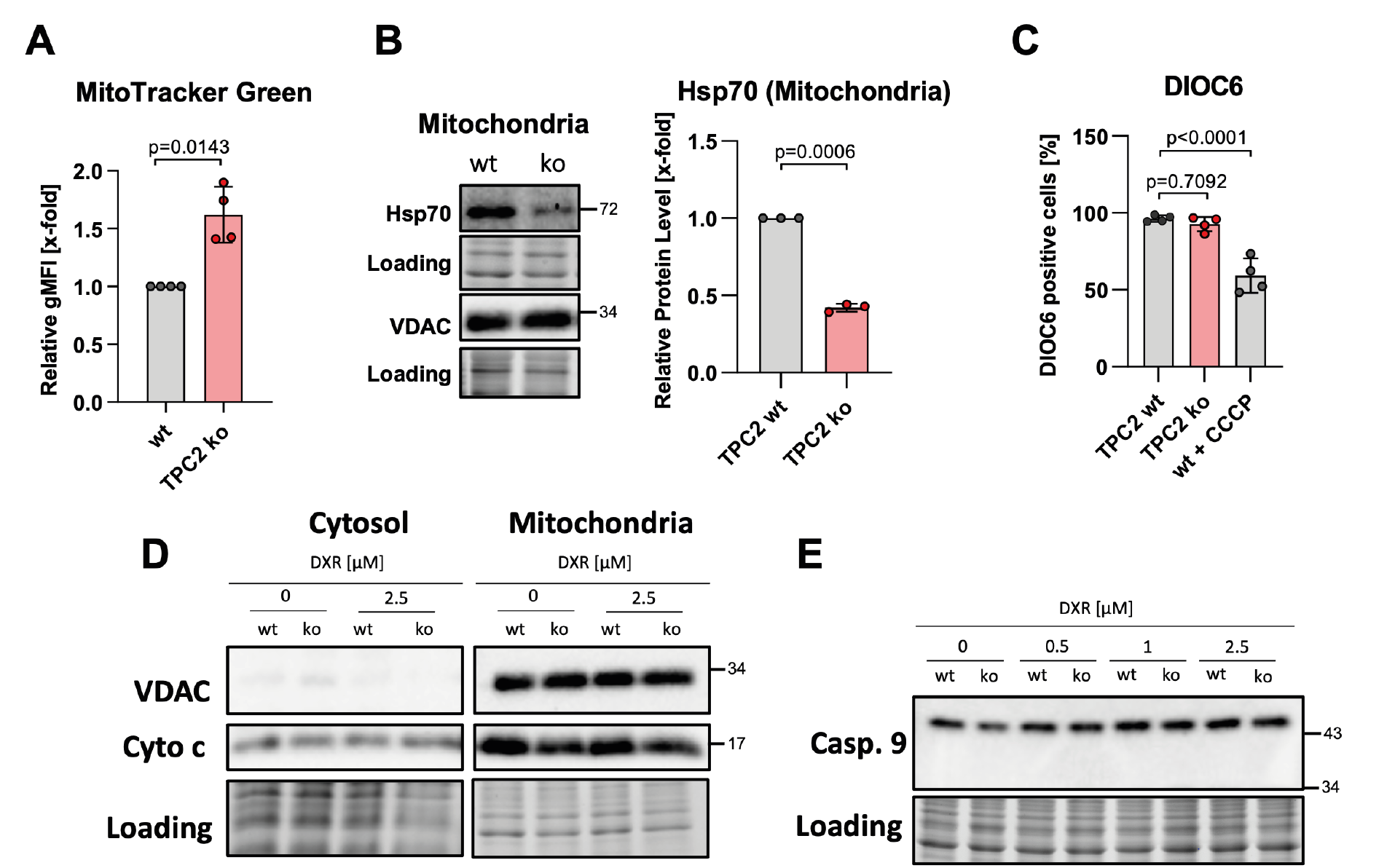

Supplement: Supplementary file 8 — Figure S7 [file 41419_2022_5105_MOESM8_ESM.png]
