# Supplementary material for: Targeting TPC2 sensitizes acute lymphoblastic leukemia cells to chemotherapeutics by impairing lysosomal function
Source: Cell Death Dis. 2022 Aug 1;13(8):668. doi: 10.1038/s41419-022-05105-z (PMC9343397; doi:10.1038/s41419-022-05105-z)
Supplement: Supplementary file 9 — Supplementary Figure Legends [file 41419_2022_5105_MOESM9_ESM.docx]

**Supplementary Figure Legends**

**Fig. S1.** **(A)** VCR-R CEM TPC2 wt and CCRF-CEM cells were treated for 72h. The antiproliferative effect of vincristine, doxorubicin and topotecan was analyzed by CellTiter-Blue assay. **(B)** Lysosomal volume was assessed by LysoTracker Green staining and flow cytometry after 4h of treatment. **(C)** Lysosomal damage was assessed by quantification of LysoTracker negative cells by flow cytometry. . **(D, E)** Expression levels of LAMP1, ATP6V0C and TFEB were analyzed by qPCR after 4h (D) or 24h (E) of treatment. Data were acquired from at least three independent experiments. Statistical significance was analyzed using the comparison of fits function of GraphPad Prism 8 **(A)**, two-way ANOVA with Sidak’s posttest **(B, C)**, or student’s t test with Welch’s correction **(D, E)**. Data are shown as mean ± SD.

**Fig. S2.** **(A-C)** Loss of TPC2 function was confirmed by endolysosomal patch clamp, nonsense-mediated decay of TPCN2 mRNA, and Sanger sequencing. **(A)** Cells were treated with 1 µM Vacuolin-1 to enlarge lysosomes and immobilized on glass slides using CellTak. Basal TPC2 currents were recorded as well as after activation with 1 µM PI(3,5)P_2_. Experiment was conducted one time. **(B)** Expression levels of TPC2 mRNA was analyzed by qPCR. Data were acquired from three independent experiments and statistical significance was analyzed using student’s t test with Welch’s correction (B). **(C)** Transfection with TPC2 sgRNA leads to insertion of a thymidine base and thus to a premature stop codon and ultimately to TPC2 loss of function. **(D)** Potential off-target sequences were predicted using the CRISPOR TEFOR in silico tool (crispor.tefor.net). All genes with a CFD score >0.0 were checked for off-targets, revealing that there are no off-targets

**Fig. S3.** G2 phase arrest was quantified after propidium iodide staining and subsequent flow cytometry after 24 h (left) and 48 h (right).

**Fig. S4.** (A, B) Relative proliferation of VCR-R CEM TPC2 wt cells was assessed by CellTiter-Blue assay after 72h of treatment. (C) Membranous P-gp levels were assessed using a FITC-coupled P-glycoprotein antibody (BD Biosciences #557002) and flow cytometry. ABCB1 mRNA level was analyzed by qPCR. **(D)** P-glycoprotein protein levels were quantified by immunoblotting. ABCB1 mRNA level was analyzed by qPCR. Membranous P-gp levels were assessed using a FITC-coupled P-glycoprotein antibody and flow cytometry. **(E)** VCR-R CEM TPC2 wt and TPC2 ko cells were treated for 72h (constant concentration of the P-gp inhibitors verapamil and ketoconazole, varying concentrations of vincristine and doxorubicin. Relative proliferation was assessed by CellTiter-Blue assay after 72h of treatment. **(F, G)** Relative proliferation of HeLa (F) and HepG2 (G) cells was assessed by CellTiter-Blue assay after 72h of treatment. Data were acquired from at least three independent experiments. Statistical significance was analyzed by two-way ANOVA with Sidak’s posttest **(A, B, F, G)**, student’s t test with Welch’s correction **(C, D)** or by using the comparison of fits function of GraphPad Prism 8 **(E)**. Data are shown as mean ± SD. **(H)** Information on PDX ALL cells used in this study. **(I)** Statistical analysis corresponding to Fig. 3G. Statistical significance was analyzed by two-way ANOVA with Sidak’s posttest. Left table shows statistical comparisons between different conditions in the group of PDX cells (middle column) and PBMCs (right column). Right table shows statistical comparisons between PDX cells and PBMCs with the same conditions.

**Fig. S5. (A)** VCR-R CEM TPC2 wt and TPC2 ko cells were treated as indicated for 4h and lysosomal volume was assessed by LysoTracker Green staining and flow cytometry. **(B)** VCR-R CEM TPC2 wt and TPC2 ko cells were treated as indicated for 72h. Relative proliferation was assessed by CellTiter-Blue assay. **(C)** Immunoblots of VCR-R CEM wt and TPC2 ko cells. Data were acquired from at least three independent experiments. Statistical significance was analyzed by two-way ANOVA with Sidak’s posttest **(A)** or by using the comparison of fits function of GraphPad Prism 8 **(B)**. Data are shown as mean ± SD.

**Fig. S6.** VCR-R CEM TPC2 wt and TPC2 ko cells were treated as indicated for 48h and lysosomal damage was assessed by quantification of LysoTracker negative cells by flow cytometry. Data were acquired from at least three independent experiments. Statistical significance was analyzed by two-way ANOVA with Sidak’s posttest. Data are shown as mean ± SD.

**Fig. S7. (A)** Mitochondrial mass was assessed by MitoTracker Green staining and flow cytometry. **(B)** Immunoblots of isolated mitochondria of VCR-R CEM TPC2 wt and TPC2 ko cells. **(C)** Mitochondrial membrane potential was assessed by DIOC6 staining and flow cytometry. CCCP served as positive control. **(D)** Immunoblots of cytosolic and mitochondrial fractions of VCR-R CEM TPC2 wt and TPC2 ko cells. **(E)** Immunoblots of VCR-R CEM TPC2 wt and TPC2 ko cells. Data were acquired from at least three independent experiments. Statistical significance was analyzed by student’s t test **(A, B)** or one-way ANOVA with Dunnet’s posttest **(C)**. Data are shown as mean ± SD.
