# Supplementary material for: Targeting TPC2 sensitizes acute lymphoblastic leukemia cells to chemotherapeutics by impairing lysosomal function
Source: Cell Death Dis. 2022 Aug 1;13(8):668. doi: 10.1038/s41419-022-05105-z (PMC9343397; doi:10.1038/s41419-022-05105-z)

Figure 2G

PARP

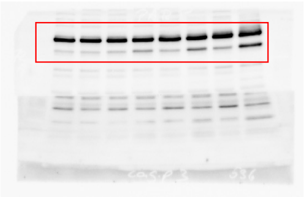

Colorimetric

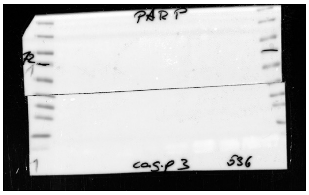

Stain-free loading control

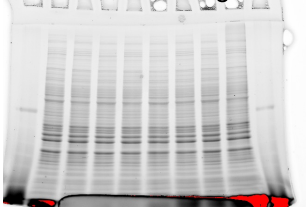

Active Caspase 3

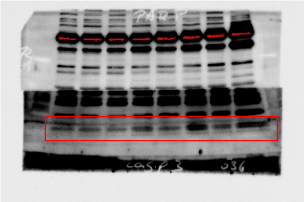

Colorimetric

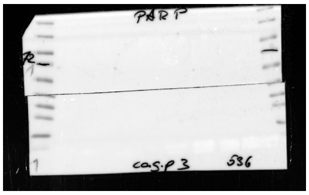

Stain-free loading control

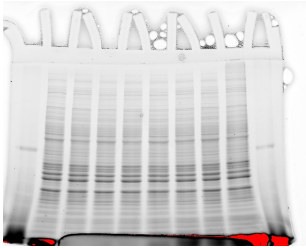

Beta Tubulin

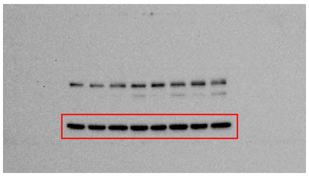

Colorimetric

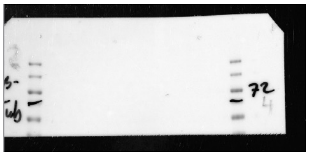

Stain-free loading control

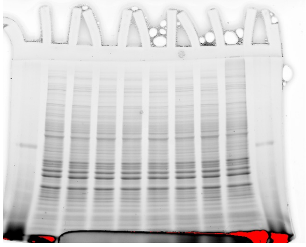

Figure 4I

γ-H2AX (Ser-139)

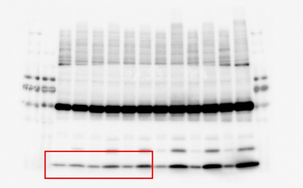

Colorimetric

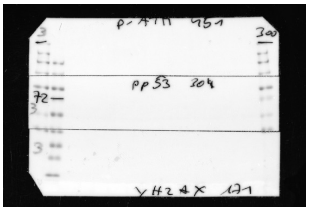

Stain-free loading control

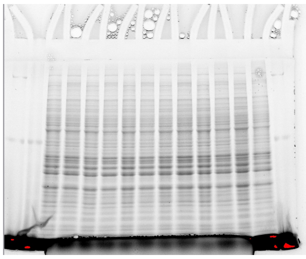

PARP

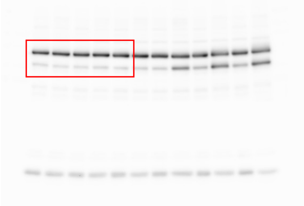

Colorimetric

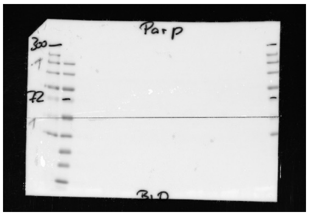

Stain-free loading control

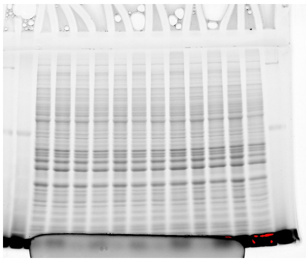

Actin

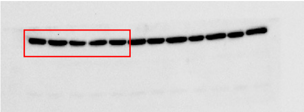

Colorimetric

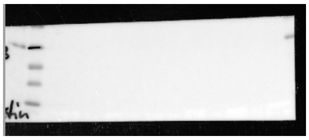

Stain-free loading control

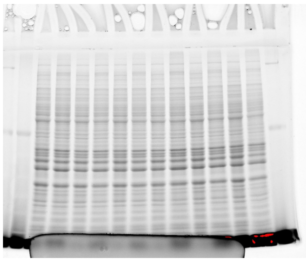

Figure 5F

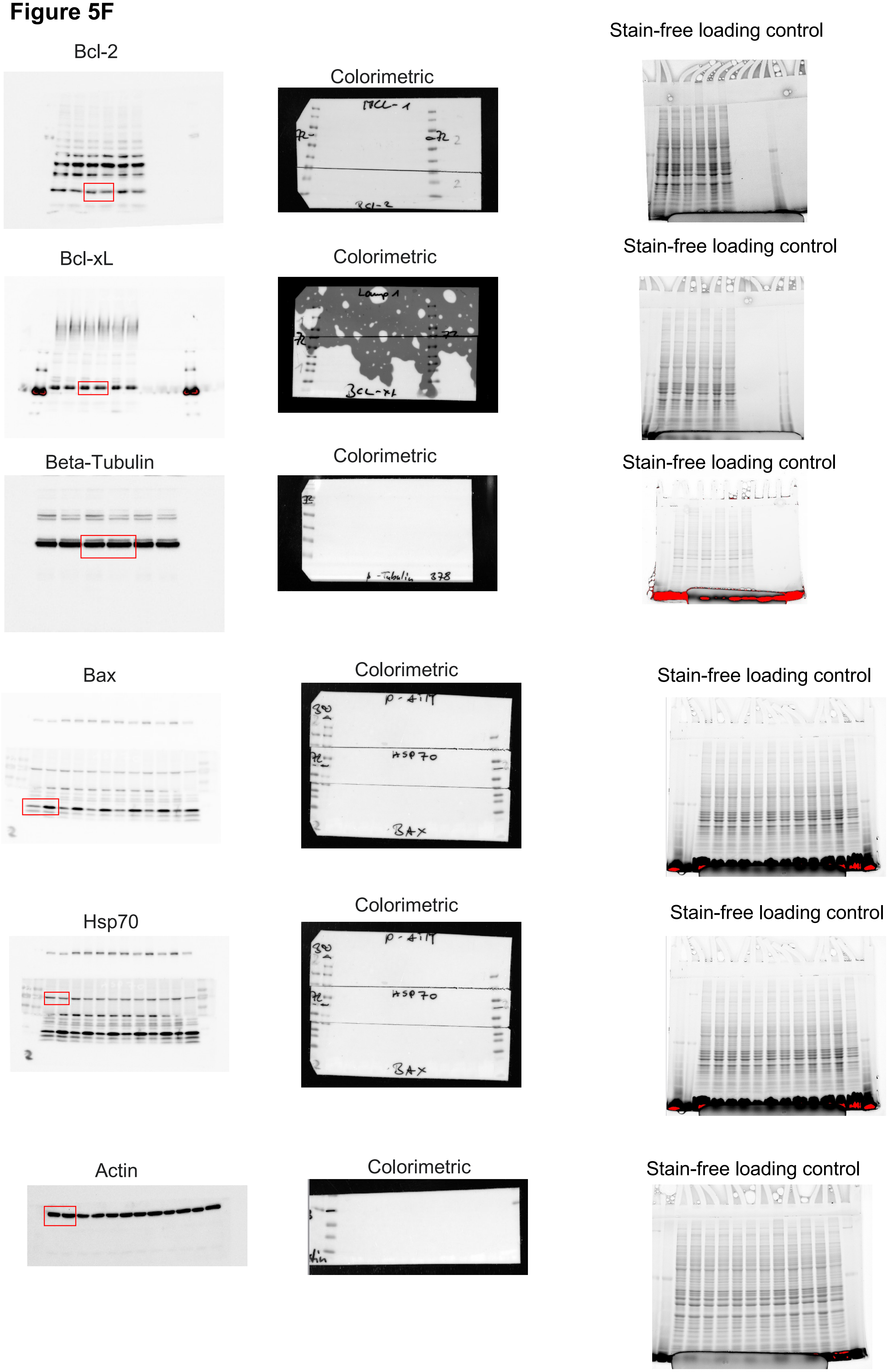
Bcl-2
Colorimetric
Stain-free loading control
Bcl-xL
Colorimetric
Stain-free loading control
Beta-Tubulin
Colorimetric
Stain-free loading control
Bax
Colorimetric
Stain-free loading control
Hsp70
Colorimetric
Stain-free loading control
Actin
Colorimetric
Stain-free loading control

Bax

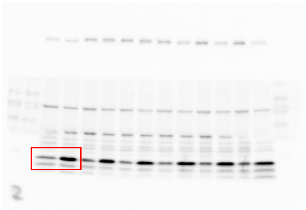

Colorimetric

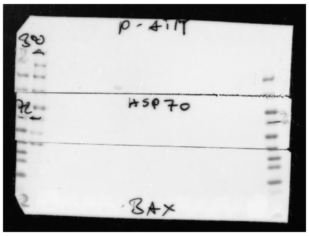

Stain-free loading control

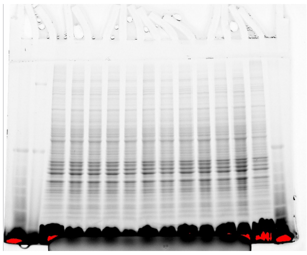

Hsp70

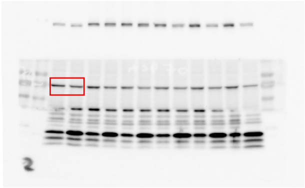

Colorimetric

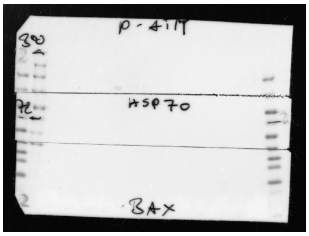

Stain-free loading control

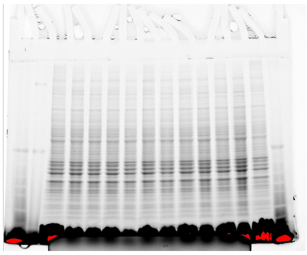

Actin

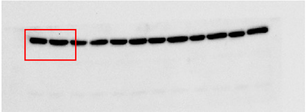

Colorimetric

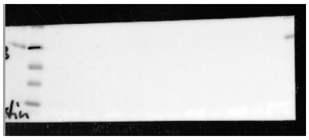

Stain-free loading control

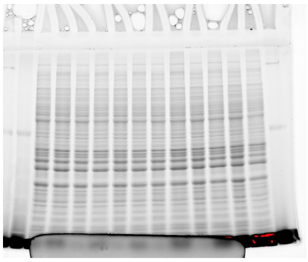

Figure 6A

Cathepsin B

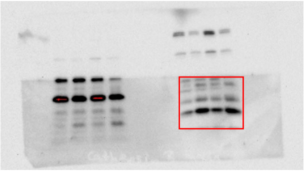

Colorimetric

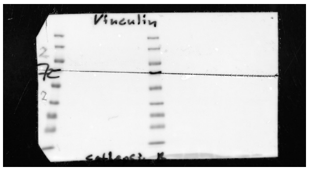

Stain-free loading control

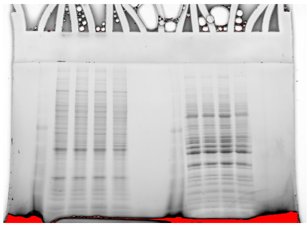

LAMP-1

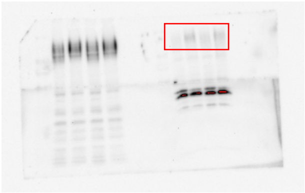

Colorimetric

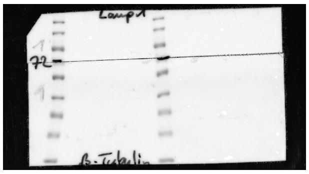

Stain-free loading control

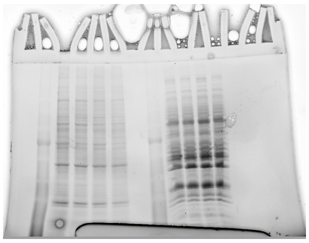

Vinculin

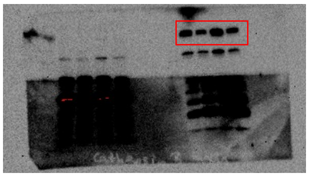

Colorimetric

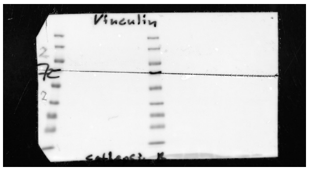

Stain-free loading control

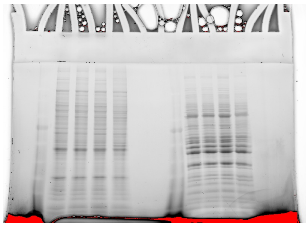

Figure 6F

Cathepsin B

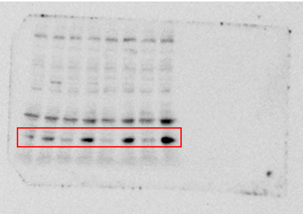

Colorimetric

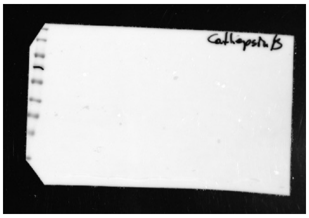

Stain-free loading control

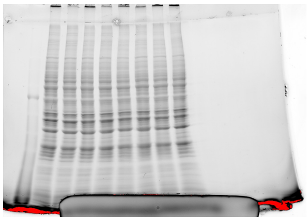

Beta Tubulin

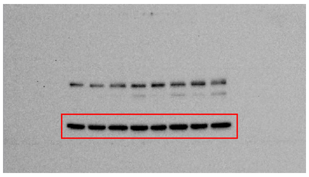

Colorimetric

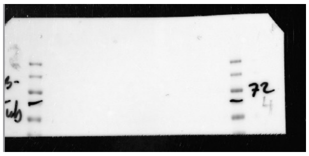

Stain-free loading control

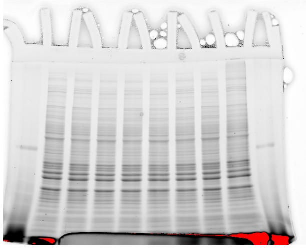

Figure 6H

Bid

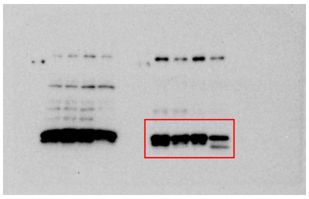

Colorimetric

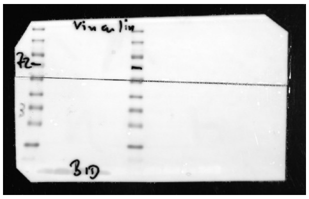

Stain-free loading control

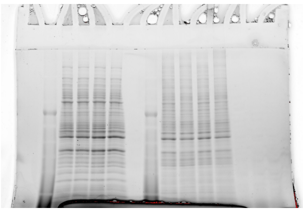

VDAC

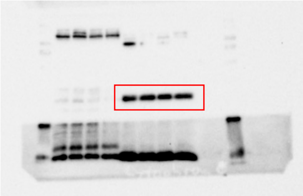

Colorimetric

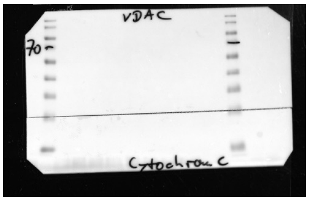

Stain-free loading control

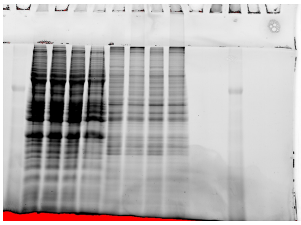

Figure 6J

Act. Caspase 3

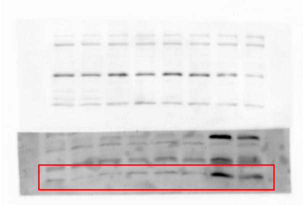

Colorimetric

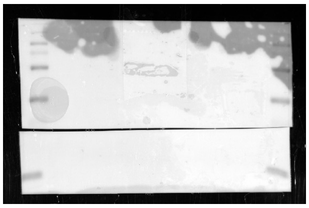

Stain-free loading control

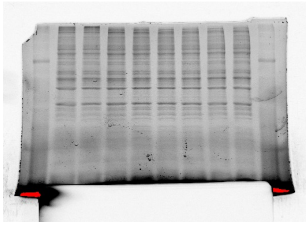

Beta Tubulin

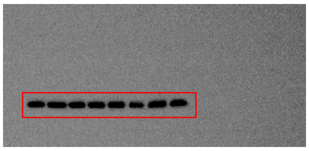

Colorimetric

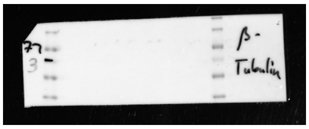

Stain-free loading control

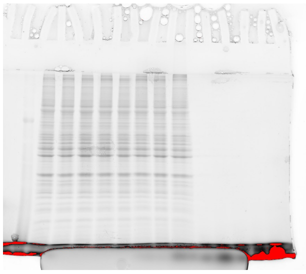

Figure S3C

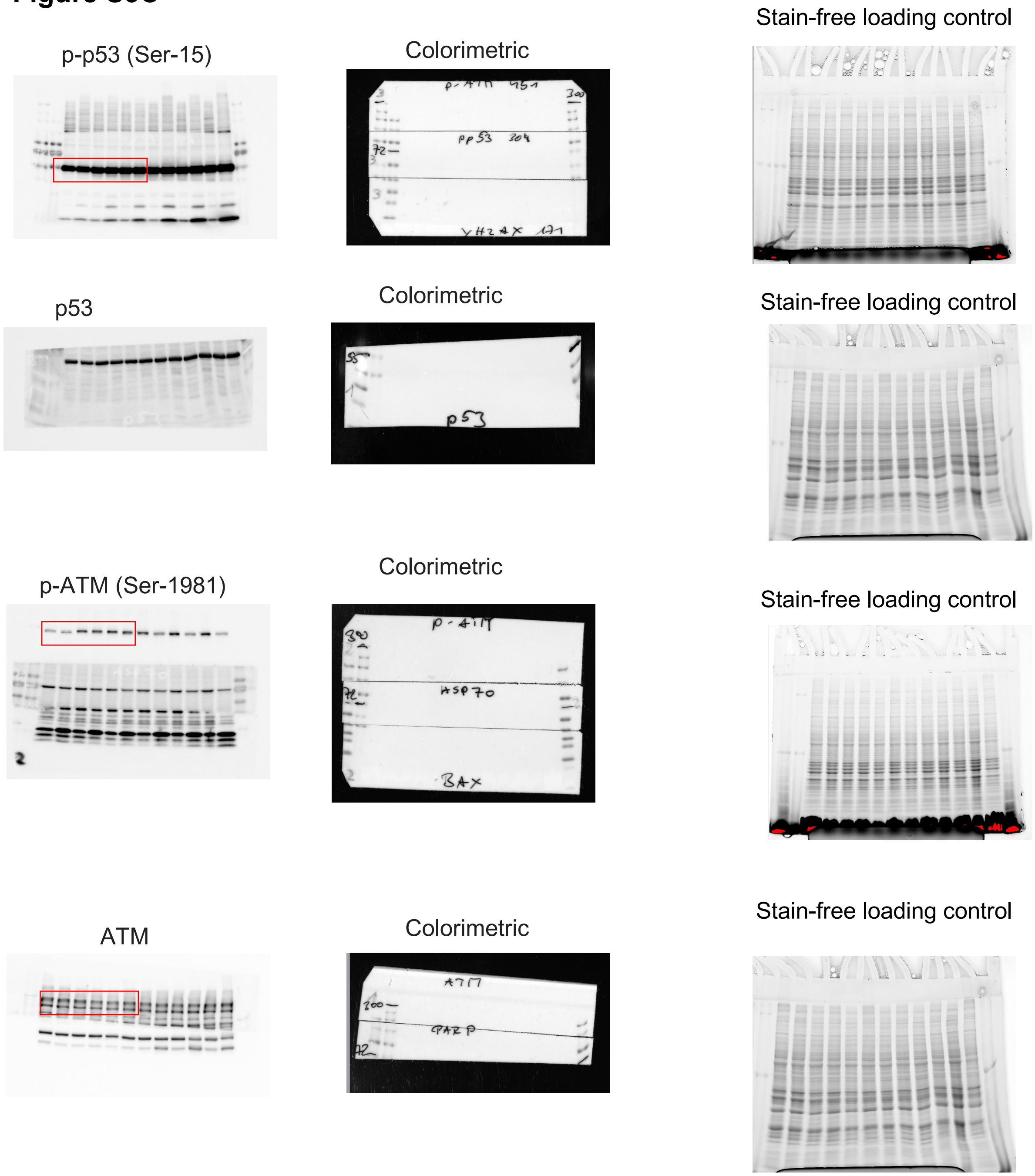

p-p53 (Ser-15)
Colorimetric
Stain-free loading control
p53
Colorimetric
Stain-free loading control
p-ATM (Ser-1981)
Colorimetric
Stain-free loading control
ATM
Colorimetric
Stain-free loading control

Figure S5B

Hsp70

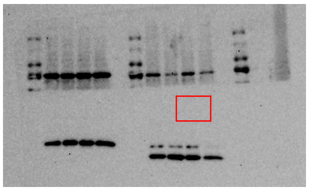

Colorimetric

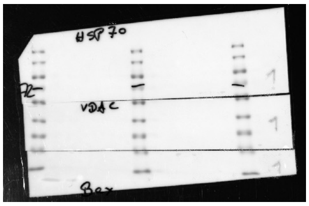

Stain-free loading control

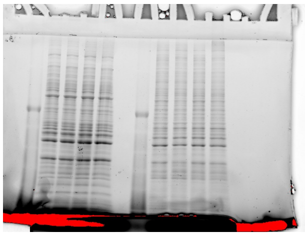

VDAC

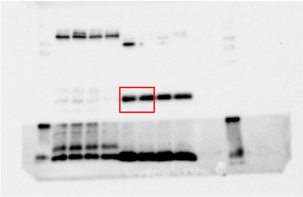

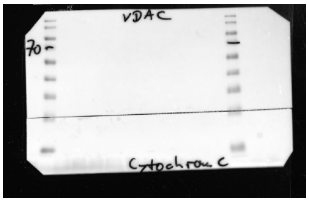

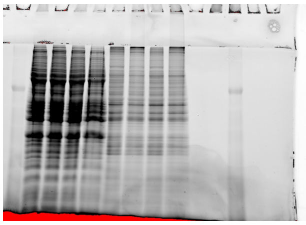

Figure S5D

VDAC

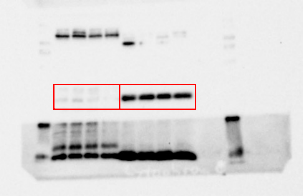

Colorimetric

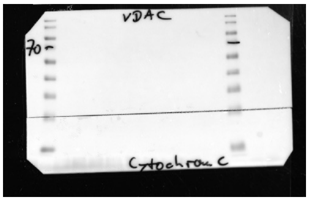

Stain-free loading control

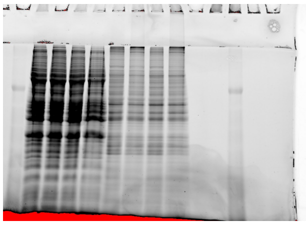

Cytochrome c

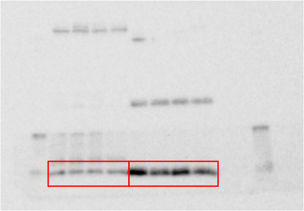

Colorimetric

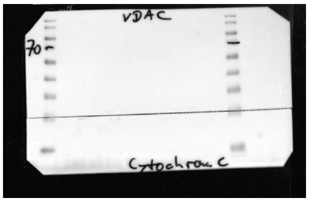

Stain-free loading control

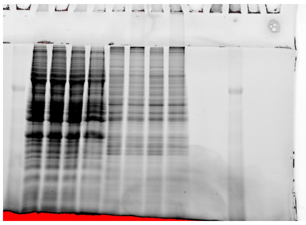

Figure S5E

Caspase 9

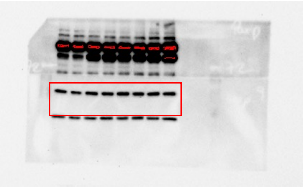

Colorimetric

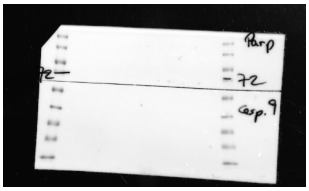

Stain-free loading control

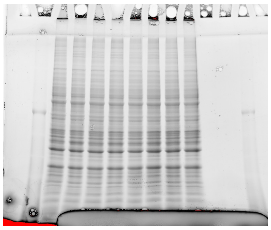

Figure 4A

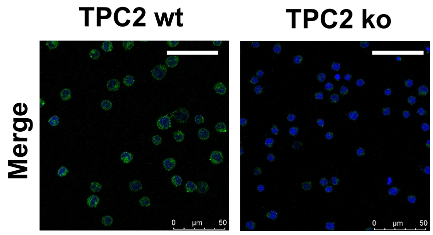

Figure 4E

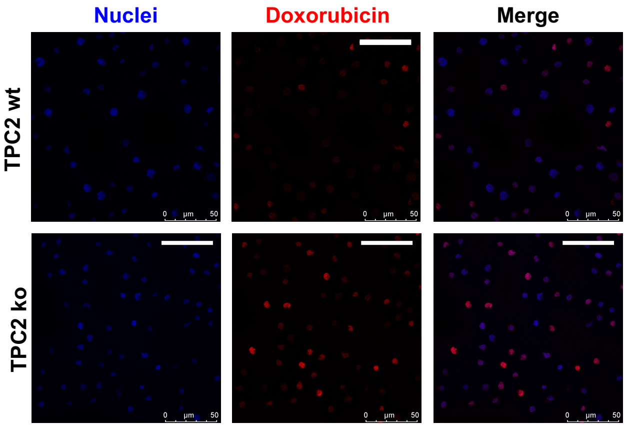

Figure 4G

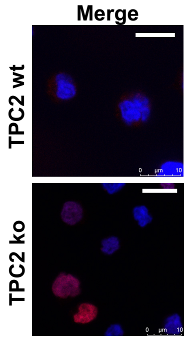

Figure 5D

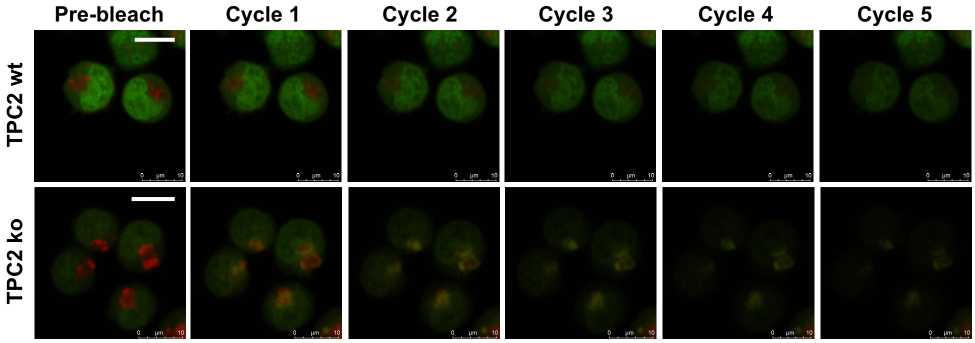

Supplement: Supplementary file 11 — Full Blots and Original Images [file 41419_2022_5105_MOESM11_ESM.pdf]
